# Association of Rurality, Race and Ethnicity, and Socioeconomic Status With the Surgical Management of Colon Cancer and Postoperative Outcomes Among Medicare Beneficiaries

**DOI:** 10.1001/jamanetworkopen.2022.29247

**Published:** 2022-08-30

**Authors:** Niveditta Ramkumar, Carrie H. Colla, Qianfei Wang, A. James O’Malley, Sandra L. Wong, Gabriel A. Brooks

**Affiliations:** 1 The Dartmouth Institute for Health Policy and Clinical Practice, Geisel School of Medicine at Dartmouth, Lebanon, New Hampshire; 2 Geisel School of Medicine at Dartmouth, Hanover, New Hampshire; 3 Congressional Budget Office, Washington, DC; 4 Department of Biomedical Data Sciences, Geisel School of Medicine at Dartmouth, Lebanon, New Hampshire; 5 Department of Surgery, Dartmouth Hitchcock Medical Center, Lebanon, New Hampshire; 6 Department of Medicine, Dartmouth Hitchcock Medical Center, Lebanon, New Hampshire

## Abstract

**Importance:**

Rural patients with colon cancer experience worse outcomes than urban patients, but the extent to which disparities are explained by social determinants is not known.

**Objectives:**

To evaluate the association of rurality with surgical treatment and outcomes of colon cancer and to investigate the intersection of rurality with race and ethnicity and socioeconomic status.

**Design, Settings, and Participants:**

This cohort study included fee-for-service Medicare beneficiaries 65 years or older diagnosed with incident, nonmetastatic colon cancer between April 1, 2016, and September 30, 2018, with follow-up until December 31, 2018. Data were analyzed from August 3, 2020, to April 30, 2021.

**Exposures:**

Rurality of patient’s residence, categorized as metropolitan, micropolitan, or small town or rural, using Rural-Urban Commuting Area codes.

**Main Outcomes and Measures:**

Receipt of surgery, emergent surgery, or minimally invasive surgery (MIS); 90-day surgical complications; and 90-day mortality.

**Results:**

Among 57 710 Medicare beneficiaries with incident, nonmetastatic colon cancer, 46.6% were men, 53.4% were women, and the mean (SD) age was 76.6 (7.2) years. In terms of race and ethnicity, 3.7% were Hispanic, 6.4% were non-Hispanic Black (hereinafter Black), 86.1% were non-Hispanic White (hereinafter White), and 3.8% were American Indian or Alaska Native, Asian or Pacific Islander, or unknown race or ethnicity. Patients residing in nonmetropolitan areas were more likely to undergo surgical resection than those residing in metropolitan areas (69.2% vs 63.9%; *P* < .001). Black race was independently associated with lower hazard of surgical resection (hazard ratio, 0.92 [95% CI, 0.88-0.95]). Race and ethnicity and measures of socioeconomic status did not modify the association of rurality with surgery. Beneficiaries from small town and rural areas had higher odds of undergoing emergent surgery (adjusted odds ratio [OR], 1.32 [95% CI, 1.20-1.44]) but lower odds of undergoing MIS (adjusted OR, 0.75 [95% CI, 0.70-0.80]), with similar findings for patients residing in micropolitan areas. Members of racial and ethnic minority groups who resided in small town and rural settings experienced higher odds of postoperative surgical complications (*P* = .001 for interaction) and mortality (*P* = .001 for interaction). Notably, White patients who resided in small town and rural areas experienced lower odds of postoperative mortality than their White metropolitan counterparts (adjusted OR, 0.81 [95% CI, 0.71-0.92]), but Black patients who resided in small town and rural areas had significantly higher odds of postoperative mortality (adjusted OR, 1.86 [95% CI, 1.16-2.97]) than their Black metropolitan counterparts.

**Conclusions and Relevance:**

These findings suggest that Medicare beneficiaries from small town and rural areas were more likely to undergo surgery for nonmetastatic colon cancer than metropolitan beneficiaries but also more likely to undergo emergent surgery and less likely to have MIS. The experiences of rural patients varied by race; rurality was associated with higher postoperative mortality for Black patients but not for other racial and ethnic groups.

## Key Points

**Question:**

Is rurality associated with receipt of surgery for nonmetastatic colon cancer?

**Findings:**

In this cohort study of 57 710 Medicare beneficiaries with colon cancer, rural patients were more likely to undergo surgery but also more likely to receive emergent surgery and less likely to have minimally invasive surgery. Black race was independently associated with lower likelihood of surgery, and the association between rurality and postoperative mortality differed by race; rurality was protective against mortality for White beneficiaries, but rural-residing Black beneficiaries had increased postoperative mortality.

**Meaning:**

Treatment and surgical outcomes in nonmetastatic colon cancer varied by rurality and by race and ethnicity, suggesting intersectional factors underlying disparities.

## Teaser

This cohort study evaluates the intersectionality of rurality, race and ethnicity, and socioeconomic status and the association of these characteristics with surgical outcomes in the treatment of nonmetastatic colon cancer among Medicare beneficiaries.

## Introduction

Health disparities between residents of urban and rural areas are well documented, suggesting that these differences arise from restricted access to health care owing to travel distance and other factors.^1,2,3,4^ Disparities in access to high-quality health care may be compounded for patients with cancer who require complex care from multiple clinicians.^4,5^

Colon cancer is one of the most common cancers in the US, with more than 100 000 new cases annually.^5,6,7^ For the 19% of the US population living in nonmetropolitan regions,^5,8^ a colon cancer diagnosis often portends worse outcomes.^4,5,9,10,11,12,13,14,15^ Nonmetastatic colon cancer can be treated by general surgeons in most rural areas.^5^ However, prior descriptive studies focusing on geography^4,5,9,10,11,12,13,14,15^ have shown that patients living in rural areas with colon cancer are less likely to undergo cancer-directed surgical treatment and more likely to die of cancer than patients living in urban areas. Little is known about the interacting effects of rurality, race and ethnicity, and socioeconomic status on the treatment and outcomes of colon cancer.^16,17^

Our objective was to evaluate the intersectionality of rurality, race and ethnicity, and socioeconomic status and the association of these characteristics with surgical outcomes in the treatment of nonmetastatic colon cancer. Outcomes such as emergent procedures and use of minimally invasive surgery (MIS) serve as markers of access to and quality of care, allowing us to study both the processes and outcomes of surgical colon cancer care by patient rurality.^18,19,20^ We hypothesized that patients living outside metropolitan areas would have less access to high-quality care, leading to inferior survival. We evaluated rurality in a national cohort of consistently insured patients for which we could separately control for race and ethnicity, socioeconomic status, and rurality.

## Methods

### Study Design and Cohort

We performed a cohort study of fee-for-service Medicare beneficiaries 65 years or older with incident, nonmetastatic colon cancer. This study was approved by the Committee for the Protection of Human Subjects at Dartmouth College, Lebanon, New Hampshire, which waived the need for informed consent because the research presented minimal risk of harm to participants and protections for privacy were in place, as required by the data use agreement. This study followed the Strengthening the Reporting of Observational Studies in Epidemiology (STROBE) reporting guideline.

We identified patients with incident colon cancer using a previously validated algorithm with minor modifications.^21,22^ We included beneficiaries meeting 1 or more of the following criteria: (1) any claim for inpatient or outpatient chemotherapy, radiotherapy, or cancer-directed surgery with a colon cancer diagnosis code; (2) 2 claims from different dates with a colon cancer diagnosis code within 12 months after a diagnostic biopsy; or (3) 2 claims with a colon cancer diagnosis code, with the first occurring within 14 days of a claim with a diagnosis code for a cancer-related symptom or complication and the second occurring within the 12 months after the cancer-related symptom. We included patients with incident colon cancer diagnoses from April 1, 2016, to September 30, 2018, allowing for minimum follow-up of 90 days until December 31, 2018.

**Figure 1.**
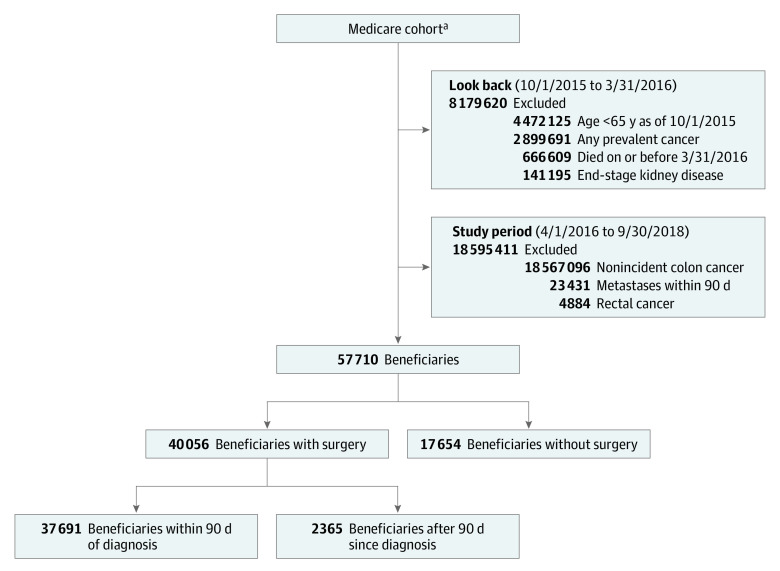
Analytic Cohorts of Incident Nonmetastatic Colon Cancer ^a^Includes all U.S.-residing fee-for-service Medicare beneficiaries with continuous enrollment in Medicare Parts A and B between October 1, 2015, and December 31, 2018 (or through death), who did not live in a nursing home (26 832 741 beneficiaries).

Among beneficiaries meeting at least 1 criterion for incident cancer, we excluded those with distant metastasis within 90 days of diagnosis (Figure 1). Evidence of distant metastasis was defined as any claim listing a diagnosis of a secondary malignant neoplasm (excepting the intra-abdominal lymph nodes). Because rectal cancer is sometimes miscoded as colon cancer in claims, we further excluded beneficiaries who had as many or more diagnoses of rectal cancer as for colon cancer within 60 days after index diagnosis (eMethods in the Supplement). The data that support the findings of this study are available with the permission from the Centers for Medicare & Medicaid Services (CMS).

### Exposures and Outcomes

The primary exposure was rurality of patient residence according to zip code plus 4-digit extensions. Levels of rurality were defined using the US Department of Agriculture 2010 Rural-Urban Commuting Area (RUCA) codes and categorized as metropolitan (RUCA 1.0-3.0), micropolitan (RUCA 4.0-6.0), and small town or rural (RUCA 7.0-10.3).

Primary outcomes included (1) receipt of cancer-directed surgery (colectomy), (2) receipt of emergent surgery, (3) receipt of MIS, (4) 90-day postoperative surgical complications, and (5) 90-day postoperative mortality. Surgical resection was assessed in the full cohort, and the remaining surgical outcomes were assessed among beneficiaries undergoing surgery within 90 days of colon cancer diagnosis.

Emergent surgery, defined as surgery that was performed within 2 calendar days of an emergency department encounter or transfer from another facility, served as a measure of both access to care and overall care coordination.^18^ Minimally invasive surgery was defined as any laparoscopic or robotic procedure according to the procedure code from *International Statistical Classification of Diseases and Related Health Problems, Tenth Revision* (*ICD-10*)*,* or *Current Procedural Terminology*. This outcome was selected as a marker of care quality because minimally invasive procedures have oncologic outcomes comparable to those for open procedures but have faster recovery times; accordingly, minimally invasive procedures are preferred when available.^20,23,24^ Surgical complications included postprocedural hemorrhage or hematoma, wound infection, anastomotic leak, and abdominal abscess.^25,26^ Secondary outcomes included receipt of adjuvant chemotherapy within 120 days of surgery, 90-day all-cause readmissions, and 90-day postoperative medical complications (including kidney failure, pulmonary compromise, acute myocardial infarction, pneumonia, venous thrombosis and pulmonary embolism, and dementia or delirium) (eMethods in the Supplement).

### Covariate Measures

All adjusted models accounted for age, sex, race and ethnicity,^27^ Medicare-Medicaid full dual eligibility (an individual-level surrogate measure for low socioeconomic status), diabetes, myocardial infarction, congestive heart failure, stroke or transient ischemic attack, chronic obstructive pulmonary disorder, end-stage kidney disease, the composite CMS–Hierarchical Condition Categories risk adjustment score,^28^ side of primary colon cancer (left, right, or unspecified), and Area Deprivation Index^29,30^ (ADI) at the zip code level. We used the Research Triangle Institute race code variable available in the CMS Master Beneficiary Summary File for race and ethnicity data. These data were collected as reported by patients from individual hospitals and further derived using the Research Triangle Institute race algorithm at CMS. We categorized race as Hispanic or non-Hispanic Black, non-Hispanic White, or other race or ethnicity. Racial categories excluded Hispanic patients unless mentioned otherwise. The ADI is a composite index of 17 US Census–based indicators used to assess local area socioeconomic deprivation created by ranking neighborhoods nationally by ADI raw score from 1 to 100, with higher scores indicating increased deprivation. The ADI was the only characteristic with missing data, missing for 2720 beneficiaries (4.7%), and we imputed the ADI for these beneficiaries (eMethods in the Supplement). Additional descriptive characteristics studied include hospital rurality, defined using the RUCA code of the hospital in which the surgery was performed, and the median distance traveled to surgical care, which was defined as the distance between zip code centroids for a patient’s residence and the hospital where they underwent surgery.

### Statistical Analysis

Data were analyzed from August 3, 2020, to April 30, 2021. We described differences in patient characteristics across rurality using proportions, means, and inferential statistics, including analysis of variance for continuous variables and χ^2^ tests for categorical variables.

Adjusted analyses consisted of 2 stages. First, we used Kaplan-Meier failure curves, log-rank tests, and multivariable Cox proportional hazards regression to assess the association of rurality with time to surgery from the initial diagnosis. Patients were censored if they died or if they did not undergo surgery by the end of the study period. Second, we used multivariable logistic regression to examine the association between rurality and surgical outcomes among patients who underwent surgery within 90 days of diagnosis. All models were adjusted for the aforementioned covariates.

To study the intersectionality of rurality, race and ethnicity, and socioeconomic status with surgical management of colon cancer, we evaluated race and ethnicity, Medicare-Medicaid dual eligibility, and ADI as effect modifiers of the association between rurality and our primary outcomes using interaction terms. We performed an exploratory univariate analysis of the association between race and ethnicity with select key demographic characteristics and outcomes stratified by rurality. We assessed statistical significance using the threshold of 2-tailed *P* < .05. All analyses were performed in Stata, version 16.1 (StataCorp LLC).

## Results

We identified 57 710 Medicare beneficiaries diagnosed with incident, nonmetastatic colon cancer from April 1, 2016, to September 30, 2018. Among this group, 46.6% were men and 53.4% were women; the mean (SD) age was 76.6 (7.2) years. In terms of race and ethnicity, 3.7% were Hispanic, 6.4% were non-Hispanic Black (hereinafter Black), 86.1% were non-Hispanic White (hereinafter White), and 3.8% were American Indian or Alaska Native, Asian or Pacific Islander, or unknown race or ethnicity. In terms of location, 74.0% lived in metropolitan areas, 12.9% lived in micropolitan areas, and 13.1% lived in small town or rural areas.

There were differences across levels of rurality in age, race and ethnicity, dual eligibility, and social deprivation (as measured by the ADI) (Table 1). Patients in small town and rural areas and micropolitan areas were marginally younger (mean [SD] age, 76.1 [6.9] years and 75.9 [6.9] years, respectively) compared with their metropolitan counterparts (mean [SD] age, 76.3 [7.1] years). Small town and rural areas had the highest proportion of dual-eligible patients (513 of 7560 [6.8%]) and the highest degree of social deprivation (median ADI, 68.0 [IQR, 53.0-80.0]), similar to that for micropolitan areas (437 of 7444 [5.9%] with dual eligibility; median ADI, 63.0 [IQR, 46.0-78.0]) but significantly different from that for metropolitan areas (2623 of 42 706 [6.1%] with dual eligibility; median ADI, 39.0 [IQR, 19.0-61.0]) (*P* = .05 for dual eligibility and *P* < .001 for ADI).

**Table 1.** Patient Characteristics of Medicare Beneficiaries With Incident Nonmetastatic Colon Cancer, by Receipt of Surgery and Rurality^a^

Characteristic	Full cohort by location (N = 57 710)				Surgical cohort by location (n = 37 691)			
	Metropolitan (n = 42 706)	Micropolitan (n = 7444)	Small town or rural (n = 7560)	*P* value	Metropolitan (n = 27 305)	Micropolitan (n = 5100)	Small town or rural (n = 5286)	*P* value
Age, mean (SD), y	76.8 (7.3)	76.3 (7.1)	76.5 (7.0)	<.001	76.3 (7.1)	75.9 (6.9)	76.1 (6.9)	<.001
Age category, y								
65-70	10 187 (23.9)	1874 (25.2)	1785 (23.6)	<.001	6853 (25.1)	1339 (26.3)	1322 (25.0)	<.001
71-75	10 038 (23.5)	1816 (24.4)	1893 (25.0)		6629 (24.3)	1284 (25.2)	1325 (25.1)	
76-80	9215 (21.5)	1674 (22.5)	1728 (22.9)		5974 (21.9)	1138 (22.3)	1229 (23.3)	
81-85	7357 (17.2)	1201 (16.1)	1208 (16.0)		4581 (16.8)	823 (16.1)	827 (15.6)	
>85	5909 (13.8)	879 (11.8)	946 (12.5)		3268 (12.0)	516 (10.1)	583 (11.0)	
Sex								
Women	22 824 (53.4)	3969 (53.4)	3997 (52.9)	.65	15 035 (55.1)	2810 (55.1)	2866 (54.2)	.51
Men	19 882 (46.6)	3475 (46.6)	3563 (47.1)		12 270 (44.9)	2290 (44.9)	2420 (45.8)	
Race and ethnicity								
Hispanic	1795 (4.2)	204 (2.7)	142 (1.9)	<.001	1125 (4.1)	131 (2.6)	88 (1.7)	<.001
Non-Hispanic Black	3034 (7.1)	356 (4.8)	305 (4.0)		1875 (6.9)	237 (4.6)	222 (4.2)	
Non-Hispanic White	36 041 (84.4)	6717 (90.2)	6915 (91.5)		23 164 (84.8)	4607 (90.3)	4834 (91.4)	
Non-Hispanic other^b^	1836 (4.3)	167 (2.2)	198 (2.6)		1141 (4.2)	125 (2.5)	142 (2.7)	
Medicare-Medicaid dual eligibility	2623 (6.1)	437 (5.9)	513 (6.8)	.05	1519 (5.6)	285 (5.6)	343 (6.5)	.03
ADI, median (IQR)^c^	39.0 (19.0-61.0)	63.0 (46.0-78.0)	68.0 (53.0-80.0)	<.001	39.0 (20.0-62.0)	63.0 (46.0-78.0)	68.0 (53.0-80.0)	<.001
Clinical characteristics								
Diabetes	9974 (23.3)	1750 (23.5)	1757 (23.2)	.93	6337 (23.2)	1215 (23.8)	1218 (23.0)	.58
MI	380 (0.9)	75 (1.0)	72 (0.9)	.57	227 (0.8)	55 (1.1)	46 (0.9)	.22
CHF	3318 (7.8)	545 (7.3)	565 (7.5)	.32	1939 (7.1)	343 (6.7)	360 (6.8)	.52
Stroke or TIA	921 (2.2)	163 (2.2)	124 (1.6)	.01	545 (2.0)	105 (2.1)	83 (1.6)	.10
COPD	3660 (8.6)	713 (9.6)	655 (8.7)	.02	2150 (7.9)	435 (8.5)	421 (8.0)	.28
End-stage liver disease	67 (0.1)	11 (0.1)	13 (0.2)	.93	27 (0.1)	<11	<11	.91
End-stage kidney disease	486 (1.1)	90 (1.2)	82 (1.1)	.77	277 (1.0)	49 (1.0)	53 (1.0)	.94
CMS-HCC risk score, median (IQR)	0.66 (0.44-1.02)	0.65 (0.44-0.98)	0.64 (0.44-0.95)	<.001	0.62 (0.41-0.97)	0.59 (0.41-0.93)	0.58 (0.43-0.89)	.02
Side of primary colon cancer								
Left	9562 (22.4)	1671 (22.4)	1782 (23.6)	<.001	6121 (22.4)	1089 (21.3)	1219 (23.1)	.007
Right	24 108 (56.5)	4505 (60.5)	4503 (59.6)		19 963 (73.1)	3815 (74.8)	3872 (73.3)	
Unspecified	9036 (21.1)	1268 (17.0)	1275 (16.9)		1221 (4.5)	196 (3.8)	195 (3.7)	

Abbreviations: ADI, Area Deprivation Index; CHF, congestive heart failure; CMS-HCC, Centers for Medicare & Medicaid Services–Hierarchical Condition Category; COPD; chronic obstructive pulmonary disease; MI, myocardial infarction; TIA, transient ischemic attack.

^a^ Unless indicated otherwise, data are expressed as No. (%) of patients. Percentages are rounded and may not total 100. Beneficiary-level rurality categories are defined using Rural-Urban Commuting Area (RUCA) codes and categorized as metropolitan (RUCA 1.0-3.0), micropolitan (RUCA 4.0-6.0), and small town and rural (RUCA 7.0-10.3).

^b^ Includes American Indian or Alaska Native, Asian or Pacific Islander, and unknown race or ethnicity.

^c^ Composite percentile measure of local socioeconomic disadvantage across zip codes plus 4-digit extension block groups using US Census–based indicators of poverty, educational level, housing, and employment. Higher numbers indicate greater deprivation.

### Receipt of Cancer-Directed Surgery

Overall, 40 056 patients with nonmetastatic colon cancer (69.4%) underwent cancer-directed surgery through December 31, 2018. Most surgical procedures (37 691 [94.1%]) occurred within 90 days of diagnosis, with very few additional operations occurring beyond 180 days (eFigure, A, in the Supplement). Beneficiaries living in micropolitan areas and small town and rural areas had similar rates of cancer-directed surgery (69.2%), significantly higher than that for beneficiaries in metropolitan areas (63.9%; *P* < .001). The median time from the claims-based diagnosis date to surgery ranged from 14 (IQR, 2-33) days among patients in small town and rural areas to 17 (IQR, 3-35) days among patients in metropolitan areas (*P* < .001).

Compared with metropolitan areas, the adjusted likelihood of undergoing surgical resection was higher in micropolitan areas (hazard ratio [HR], 1.05 [95% CI, 1.02-1.08]) and small town and rural areas (HR, 1.08 [95% CI, 1.05-1.12]) (Table 2). Black patients were less likely to undergo surgery than White patients (HR, 0.92 [95% CI, 0.88-0.95]). Further, race and ethnicity, dual eligibility, and ADI did not significantly modify the association between rurality and receipt of cancer-directed surgery (eFigure, B, in the Supplement).

**Table 2.** Estimated Primary Outcomes From Fully Adjusted Regression Models^a^

Covariate	Receipt of surgery, overall cohort (N = 57 710)		Surgical cohort (n = 37 691)							
			Emergent surgery		MIS		Surgical complications		Mortality	
	HR (95% CI)	*P* value	OR (95% CI)	*P* value	OR (95% CI)	*P* value	OR (95% CI)	*P* value	OR (95% CI)	*P* value
Region										
Metropolitan	1 [Reference]	NA	1 [Reference]	NA	1 [Reference]	NA	1 [Reference]	NA	1 [Reference]	NA
Micropolitan	1.05 (1.02-1.08)	<.001	1.25 (1.14-1.37)	<.001	0.70 (0.66-0.75)	<.001	1.09 (0.99-1.20)	.08	0.96 (0.86-1.08)	.52
Small town or rural	1.09 (1.05-1.12)	<.001	1.32 (1.20-1.44)	<.001	0.75 (0.70-0.80)	<.001	1.08 (0.99-1.19)	.09	0.88 (0.78-0.99)	.03
Race and ethnicity										
Hispanic	0.96 (0.91-1.01)	.11	1.03 (0.87-1.23)	.70	1.02 (0.91-1.14)	.73	1.11 (0.95-1.31)	.19	0.99 (0.81-1.22)	.95
Non-Hispanic Black	0.92 (0.88-0.95)	<.001	1.00 (0.88-1.15)	.95	0.93 (0.85-1.02)	.12	1.03 (0.90-1.17)	.69	1.04 (0.89-1.22)	.60
Non-Hispanic White	1 [Reference]	NA	1 [Reference]	NA	1 [Reference]	NA	1 [Reference]	NA	1 [Reference]	NA
Non-Hispanic other	0.95 (0.91-1.01)	.08	1.03 (0.87-1.23)	.72	1.21 (1.08-1.35)	<.001	1.01 (0.85-1.19)	.92	0.81 (0.64-1.03)	.08
Full dual eligibility (vs not)	0.97 (0.93-1.01)	.14	1.44 (1.27-1.64)	<.001	0.87 (0.79-0.96)	<.001	1.14 (1.00-1.30)	.05	1.30 (1.12-1.51)	<.001
ADI, every 10 ranks	1.00 (1.00-1.01)	<.001	1.03 (1.02-1.05)	<.001	0.93 (0.92-0.94)	<.001	1.02 (1.01-1.03)	.01	1.07 (1.05-1.08)	<.001
Age category, y										
65-70	1 [Reference]	NA	1 [Reference]	NA	1 [Reference]	NA	1 [Reference]	NA	1 [Reference]	NA
71-75	1.00 (0.97-1.03)	.98	1.11 (1.00-1.23)	.05	1.00 (0.94-1.06)	.98	1.04 (0.94-1.16)	.42	1.31 (1.10-1.57)	<.001
76-80	0.97 (0.94-1.00)	.05	1.37 (1.24-1.51)	<.001	0.89 (0.84-0.95)	<.001	1.04 (0.94-1.15)	.47	1.95 (1.64-2.31)	<.001
81-85	0.97 (0.94-1.00)	.09	1.73 (1.55-1.92)	<.001	0.73 (0.68-0.78)	<.001	1.02 (0.91-1.14)	.76	2.64 (2.23-3.13)	<.001
>85	0.90 (0.86-0.93)	<.001	2.97 (2.66-3.31)	<.001	0.54 (0.50-0.59)	<.001	1.03 (0.92-1.16)	.57	4.26 (3.61-5.02)	<.001
Female sex	1.05 (1.03-1.07)	<.001	1.11 (1.00-1.23)	.05	0.97 (0.93-1.02)	.22	0.89 (0.84-0.95)	<.001	0.81 (0.75-0.88)	<.001
Side of colon cancer										
Left	1 [Reference]	NA	1 [Reference]	NA	1 [Reference]	NA	1 [Reference]	NA	1 [Reference]	NA
Right	1.65 (1.61-1.69)	<.001	0.79 (0.73-0.85)	<.001	1.33 (1.26-1.39)	<.001	0.77 (0.72-0.83)	<.001	1.11 (1.02-1.22)	.02
Unspecified	0.17 (0.16-0.18)	<.001	0.92 (0.78-1.08)	.31	0.99 (0.89-1.11)	.87	1.01 (0.86-1.18)	.93	0.27 (0.23-0.32)	<.001
Diabetes	1.02 (1.00-1.05)	.11	0.76 (0.70-0.83)	<.001	1.04 (0.98-1.09)	.19	1.08 (0.99-1.16)	.07	1.00 (0.91-1.10)	.96
MI	1.09 (0.97-1.21)	.14	0.88 (0.62-1.26)	.49	1.12 (0.89-1.41)	.34	0.98 (0.72-1.35)	.92	0.94 (0.67-1.31)	.71
CHF	1.05 (1.00-1.10)	.06	0.91 (0.79-1.05)	.21	1.03 (0.93-1.13)	.62	1.11 (0.97-1.28)	.14	1.08 (0.94-1.25)	.29
Stroke or TIA	1.10 (1.03-1.19)	.01	0.93 (0.74-1.18)	.55	1.06 (0.91-1.24)	.47	0.99 (0.79-1.23)	.92	0.88 (0.69-1.13)	.33
COPD	0.99 (0.96-1.04)	.80	1.03 (0.91-1.17)	.61	0.93 (0.85-1.01)	.08	1.00 (0.88-1.13)	>.99	1.41 (1.24-1.60)	<.001
End-stage kidney disease	0.90 (0.81-0.99)	.03	1.22 (0.91-1.63)	.18	0.82 (0.66-1.01)	.06	1.13 (0.85-1.51)	.39	1.58 (1.21-2.07)	<.001
CMS-HCC risk score	0.86 (0.84-0.88)	<.001	1.11 (1.03-1.19)	.01	0.88 (0.84-0.92)	<.001	1.15 (1.07-1.23)	<.001	1.00 (0.91-1.10)	.96

Abbreviations: ADI, Area Deprivation Index; CHF, congestive heart failure; CMS-HCC, Centers for Medicare & Medicaid Services–Hierarchical Condition Category; COPD; chronic obstructive pulmonary disease; HR, hazard ratio; MI, myocardial infarction; MIS, minimally invasive surgery; NA, not applicable; OR, odds ratio; TIA, transient ischemic attack.

^a^ Models were adjusted for age, sex, race and ethnicity, Medicare-Medicaid dual eligibility, ADI, cancer side, diabetes, CHF, COPD, previous MI, previous stroke or TIA, end-stage kidney disease, and CMS-HCC score.

### Surgical Management and Outcomes After Cancer-Directed Surgery

Of 57 710 beneficiaries with incident, nonmetastatic colon cancer, 37 691 (65.3%) underwent surgical resection within 90 days of diagnosis. Minimally invasive surgery accounted for 21 710 (57.6%) nonemergent colectomy procedures. Patients living in metropolitan areas underwent surgery a median of 7.1 (IQR, 3.4-14.2) miles from their place of residence, and 26 201 (97.7%) underwent surgery in a metropolitan hospital. Patients living in micropolitan areas and small town and rural areas underwent surgery a median of 14.9 (IQR, 0-37.2) and 32.2 (IQR, 17.3-56.1) miles from their place of residence, respectively. Most patients from small town and rural areas underwent surgery in a metropolitan hospital (2983 [57.6%]).

The odds of emergent surgery were significantly higher for residents of micropolitan areas (unadjusted OR, 1.27 [95% CI, 1.16-1.39]; adjusted OR, 1.25 [95% CI, 1.14-1.37]) and small town and rural areas (unadjusted OR, 1.39 [95% CI, 1.28-1.51]; adjusted OR, 1.32 [95% CI, 1.20-1.44]), compared with residents of metropolitan areas (Table 3). Residents of micropolitan areas (unadjusted OR, 0.63 [95% CI, 0.59-0.66]; adjusted OR, 0.70 [95% CI, 0.66-0.75]) and small town and rural areas (unadjusted OR, 0.64 [95% CI, 0.60-0.68]; adjusted OR, 0.75 [95% CI, 0.70-0.80]) were also significantly less likely to undergo MIS. When limited to only nonemergent procedures, MIS was still less likely among patients residing in micropolitan areas (OR, 0.71 [95% CI, 0.66-0.76]) and small town and rural areas (OR, 0.76 [95% CI, 0.71-0.81]). Unadjusted analyses showed increased odds of postoperative surgical complications for patients residing in nonmetropolitan areas (OR for micropolitan, 1.12 [95% CI, 1.02-1.22]; OR for small town and rural, 1.12 [95% CI, 1.03-1.23]); however, these differences were not statistically significant in adjusted analyses. Unadjusted analysis showed no statistically significant geographic differences in 90-day postoperative mortality, whereas the adjusted analysis showed significantly lower mortality for residents of small town and rural areas compared with residents of metropolitan areas (adjusted OR, 0.88 [95% CI, 0.78-0.99]). Medicare-Medicaid dual eligibility and higher ADI were independently associated with less MIS (ORs, 0.87 [95% CI, 0.79-0.96] and 0.93 [95% CI, 0.92-0.94], respectively), more emergent operations (ORs, 1.44 [95% CI, 1.27-1.64] and 1.03 [95% CI, 1.02-1.05], respectively), more postoperative surgical complications (ORs, 1.14 [95% CI, 1.00-1.30] and 1.02 [95% CI, 1.01-1.03], respectively), and greater 90-day postoperative mortality (ORs, 1.30 [95% CI, 1.12-1.51] and 1.07 [95% CI, 1.05-1.08]); however, race and ethnicity were not consistently associated with adverse outcomes among surgically treated patients (Table 2).

**Table 3.** Association of Rurality With Surgical Treatment and Outcomes Among Medicare Beneficiaries Undergoing Surgery Within 90 Days of Diagnosis for Incident Nonmetastatic Colon Cancer

Outcome	Rate by region, No. (%)	Overall rate, No. (%) (N = 37 691)	Unadjusted		Adjusted^a^	
			OR (95% CI)	*P* value	OR (95% CI)	*P* value
Emergent surgery						
Metropolitan	2993 (11.0)	4456 (11.8)	1 [Reference]	NA	1 [Reference]	NA
Micropolitan	691 (13.5)		1.27 (1.16-1.39)	<.001	1.25 (1.14-1.37)	<.001
Small town or rural	772 (14.6)		1.39 (1.28-1.51)	<.001	1.32 (1.20-1.44)	<.001
Minimally invasive surgery						
Metropolitan	16 574 (60.7)	21 710 (57.6)	1 [Reference]	NA	1 [Reference]	NA
Micropolitan	2507 (49.1)		0.63 (0.59-0.66)	<.001	0.70 (0.66-0.75)	<.001
Small town or rural	2629 (49.7)		0.64 (0.60-0.68)	<.001	0.75 (0.70-0.80)	<.001
Postoperative surgical complications^b^						
Metropolitan	3115 (11.4)	4424 (11.7)	1 [Reference]	NA	1 [Reference]	NA
Micropolitan	641 (12.6)		1.12 (1.02-1.22)	.02	1.09 (0.99-1.20)	.08
Small town or rural	668 (12.6)		1.12 (1.03-1.23)	.01	1.08 (0.99-1.19)	.09
Postoperative mortality						
Metropolitan	2064 (7.5)	2827 (7.5)	1 [Reference]	NA	1 [Reference]	NA
Micropolitan	389 (7.6)		1.09 (0.97-1.21)	.15	0.96 (0.86-1.08)	.52
Small town or rural	374 (7.1)		1.02 (0.92-1.15)	.67	0.88 (0.78-0.99)	.03
Postoperative adjuvant chemotherapy						
Metropolitan	3308 (12.1)	4540 (12.0)	1 [Reference]	NA	1 [Reference]	NA
Micropolitan	614 (12.0)		1.07 (0.98-1.17)	.14	0.97 (0.88-1.06)	.48
Small town or rural	618 (11.7)		1.06 (0.97-1.16)	.20	0.97 (0.88-1.07)	.58
Postoperative medical complications^c^						
Metropolitan	7989 (29.3)	10 962 (29.1)	1 [Reference]	NA	1 [Reference]	NA
Micropolitan	1494 (29.3)		1.00 (0.94-1.07)	.96	0.97 (0.90-1.04)	.33
Small town or rural	1479 (28.0)		0.94 (0.88-1.00)	.06	0.89 (0.82-0.95)	<.001
Postoperative all-cause readmission						
Metropolitan	4700 (17.2)	6561 (17.4)	1 [Reference]	NA	1 [Reference]	NA
Micropolitan	924 (18.1)		1.06 (0.98-1.15)	.12	1.04 (0.95-1.12)	.40
Small town or rural	937 (17.7)		1.04 (0.96-1.12)	.37	1.00 (0.92-1.09)	.93

Abbreviations: NA, not applicable; OR, odds ratio.

^a^ Models were adjusted for age, sex, race and ethnicity, Medicare-Medicaid dual eligibility, area deprivation index, cancer side, diabetes, congestive heart failure, chronic obstructive pulmonary disease, previous myocardial infarction, stroke or transient ischemic attack, end-stage kidney disease, and Centers for Medicare & Medicaid Services–Hierarchical Condition Category score.

^b^ Includes postprocedural hemorrhage or hematoma, wound infection, anastomotic leak, and abdominal abscess.

^c^ Includes kidney failure, pulmonary compromise, acute myocardial infarction, pneumonia, venous thrombosis and pulmonary embolism, and dementia or delirium.

After adjustment, rurality was not associated with secondary outcomes of postoperative medical complications, receipt of adjuvant chemotherapy, or 90-day all-cause readmission (Table 3). The exception was lower adjusted odds of postoperative medical complications for patients from small town and rural areas (OR, 0.89 [95% CI, 0.82-0.95]) (Table 3).

### Intersectionality of Rurality With Socioeconomic Status and Race and Ethnicity

**Figure 2.**
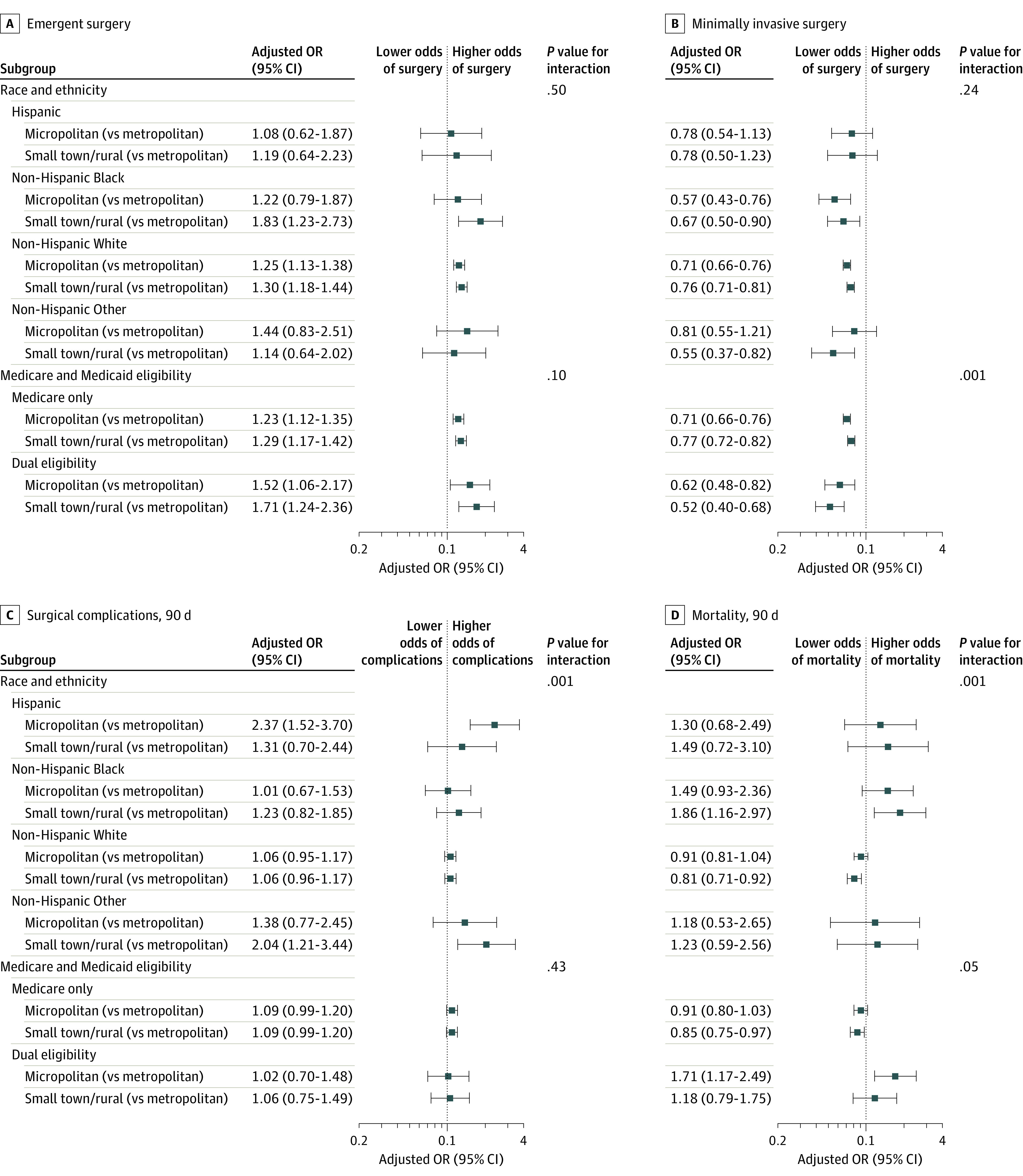
Forest Plot of Adjusted Odds Ratios (ORs) for Primary Outcomes by Subgroups of Race and Ethnicity and Medicare-Medicaid Dual Eligibility

Race and ethnicity constituted a modifier for the association of rurality with surgical complications (*P* = .001 for interaction) and postoperative mortality (*P* = .001 for interaction) but not for receipt of emergent surgery or MIS (Figure 2). The odds of a postoperative surgical complication were more than double for micropolitan-residing Hispanic patients (adjusted OR, 2.37 [95% CI, 1.52-3.70]) than for Hispanic patients in metropolitan areas. Although White patients living in small town and rural areas had lower adjusted odds of postoperative mortality than White patients in metropolitan areas (adjusted OR, 0.81 [95% CI, 0.71-0.92]), Black patients in small town and rural areas had nearly twice the odds of postoperative mortality when compared with Black patients in metropolitan areas (adjusted OR, 1.86 [95% CI, 1.16-2.97]).

Patients with Medicare-Medicaid dual eligibility in micropolitan areas and small town and rural areas were less likely to undergo minimally invasive procedures than non–dual eligible beneficiaries in the same level of rurality (*P* < .001 for interaction). Dual eligibility did not modify the association of rurality with emergent surgery or postoperative surgical complications (Figure 2).

### Association of Race and Ethnicity With Rurality

Patients from underrepresented racial and ethnic groups were 4 to 8 times more likely to have Medicare-Medicaid dual eligibility than White patients at each level of rurality (eTable in the Supplement). Across rurality, Black and Hispanic beneficiaries lived in more deprived areas than White beneficiaries; for example, in rural areas, the median ADI was 83.0 (IQR, 70.0-92.0) for Black beneficiaries, 75.5 (IQR, 60.8-89.0) for Hispanic beneficiaries, 67.0 (IQR, 52.1-79.2) for White beneficiaries, and 73.8 (IQR, 55.0-86.0) for beneficiaries of other races and ethnicities (*P* < .001).

## Discussion

In this study of fee-for-service Medicare beneficiaries with incident nonmetastatic colon cancer, we found that the rurality of a patient’s residence was associated with surgical management and postoperative outcomes in unexpected ways: patients residing in small town and rural areas were more likely to undergo surgery than those from metropolitan areas but were also more likely to undergo emergent surgery and less likely to have MIS. Furthermore, important differences in postoperative outcomes by rurality became apparent after stratifying by race and ethnicity, noting that rurality was associated with higher postoperative mortality for Black patients but not for other racial and ethnicity groups. Our findings highlight the intersectional nature of underlying disparities in colon cancer management.^31^

Prior studies^4,14^ have suggested that the medical and surgical resources to treat colorectal cancer were less likely to be found in rural areas, resulting in poor geographic access to specialty care, long travel distances for nonmetropolitan patients, reduced uptake of recommended services, and inferior survival rates. However, we found that patients who resided in nonmetropolitan areas were more likely to undergo surgery than patients from metropolitan areas. This finding seemingly refutes the notion of reduced access to surgical treatment of colon cancer in nonmetropolitan areas, and concerns about differences in timeliness and quality of care across levels of rurality require further examination. Patients residing in micropolitan areas and in small town and rural areas had a shorter median time to surgery compared with patients from metropolitan areas. Much of the literature suggests that there are delays in care for patients who reside in rural areas, but emerging evidence suggests otherwise.^14^ We observed more patients from rural areas undergoing emergent surgery, which influences the shorter window to surgery for these patients but is not necessarily consistent with better overall care. Wait times for services provided locally may be shorter in smaller community settings and could explain shorter time to surgery in rural settings. Alternatively, delays in care may occur before the time window we observed, mainly in the form of delayed diagnoses and longer time elapsed from onset of symptoms to surgery.

Although poorer surgical outcomes have been reported for patients residing in rural areas^14^ and Black patients,^32,33,34,35^ to our knowledge, our study is among the first to explore the intersection of these 2 factors. Our findings corroborate that patient rurality of residence was independently associated with higher odds of emergent surgery, lower odds of undergoing a minimally invasive procedure, and higher odds of postoperative mortality. When considering the intersecting role of rurality and race and ethnicity, we found that associations between rurality and indicators of care quality such as emergent surgery and MIS were not different across racial and ethnic groups. However, there were racial and ethnic differences in the association of rurality and 90-day postoperative mortality. Among White patients, rural living was associated with a lower adjusted odds of mortality (compared with metropolitan residence), whereas for Black patients, rural living was associated with a higher adjusted odds of mortality. We noted several differences in the characteristics of White vs Black rural-residing patients undergoing operations: Black patients were more likely to live in an area of higher deprivation and were more likely to have Medicare-Medicaid dual eligibility, indicating socioeconomic deprivation or disability. Although we adjusted for these factors, it appears that the experience of health care in rural settings is different for Black and White beneficiaries, suggesting the role of interpersonal and structural barriers (eg, discrimination, mistrust, structural racism) that contribute to this racial disparity.^36,37^

### Limitations

Our study has some limitations. First, the study cohort consisted of a federally insured population of patients 65 years and older; thus, our findings might not be generalizable to younger patients or to those who are uninsured. We excluded patients younger than 65 years to match the predominant and most generalizable population captured in the Medicare data, because beneficiaries younger than 65 years qualify for Medicare for specific statutory reasons (eg, end-stage kidney disease) and are not representative of the general population of younger patients. Second, although we aimed to form a cohort of patients with nonmetastatic colon cancer, some misclassification may have occurred owing to underreporting of diagnosis codes for secondary malignant neoplasms (metastasis). The proportion of patients undergoing surgery in our cohort was similar to that described in the existing literature (approximately 70%), and the proportion of patients with metastatic disease identified in our incident cancer cohort (approximately 24%) aligns with previous Medicare studies, suggesting that misclassification of patients with metastatic cancer is likely small.^32^ Third, we inferred diagnosis date from claims based on the first appearance of the relevant *ICD-10* diagnosis code for colon cancer. However, there may be discrepancies between when the diagnosis was suspected and when it was ultimately coded, a lag time that could vary across regions.

Using 100% fee-for-service Medicare claims data files allowed us to study a large cohort of more than 10 000 patients with nonmetastatic colon cancer undergoing surgery in nonmetropolitan areas. However, more than 90% of patients in rural areas were White, and the number of patients from minoritized racial and ethnic groups in the surgical study cohort was relatively small. Although we identified key associations of surgical management of colon cancer with rurality and race and ethnicity, we were unable to evaluate some sources of disparities owing to limited power.

## Conclusions

The findings of this cohort study suggest that Medicare beneficiaries from micropolitan areas and small town and rural areas had inconsistent outcomes in terms of access and quality of care for nonmetastatic colon cancer. They were more likely to undergo surgery for nonmetastatic colon cancer than metropolitan beneficiaries but were also more likely to undergo emergent surgery and less likely to have MIS. The experiences of patients who reside in rural areas appear to vary by race and ethnicity in clinically meaningful ways; rurality was protective against postoperative mortality for White beneficiaries, but for Black beneficiaries rurality was associated with increased postoperative mortality. These findings call for considering a multidimensional characterization of rural patients to account for the compounding role of individual and area-level sociodemographic risk factors to address health disparities associated with rurality, race and ethnicity, and socioeconomic status.

## Notes

Supplement. **eMethods.** Cohort Definition and Diagnosis and Procedure Codes **eFigure.** Time to Receipt of Surgery in the Overall Cohort (N = 57 710) and by Rurality **eTable.** Patient Characteristics and Surgical Management Measures Among the Surgical Cohort by Rurality and by Race and Ethnicity
