# Supplementary material for: Association of Rurality, Race and Ethnicity, and Socioeconomic Status With the Surgical Management of Colon Cancer and Postoperative Outcomes Among Medicare Beneficiaries
Source: JAMA Netw Open. 2022 Aug 30;5(8):e2229247. doi: 10.1001/jamanetworkopen.2022.29247 (PMC9428741; doi:10.1001/jamanetworkopen.2022.29247)
Supplement: Supplement. — eMethods. Cohort Definition and Diagnosis and Procedure Codes eFigure. Time to Receipt of Surgery in the Overall Cohort (N = 57 710) and by Rurality eTable. Patient Characteristics and Surgical Management Measures Among the Surgical Cohort by Rurality and by Race and Ethnicity [file jamanetwopen-e2229247-s001.pdf]

Supplementary Online Content

Ramkumar N, Colla CH, Wang Q, O'Malley AJ, Wong SL, Brooks GA. Association of rurality, race and ethnicity, and socioeconomic status with the surgical management of colon cancer and postoperative outcomes among Medicare beneficiaries. *JAMA Netw Open*. 2022;5(8):e2229247. doi:10.1001/jamanetworkopen.2022.29247

eMethods. Cohort Definition and Diagnosis and Procedure Codes
eFigure. Time to Receipt of Surgery in the Overall Cohort (N = 57 710) and by Rurality
eTable. Patient Characteristics and Surgical Management Measures Among the Surgical Cohort, by Rurality and by Race and Ethnicity

This supplementary material has been provided by the authors to give readers additional information about their work.

© 2022 Ramkumar N et al. *JAMA Network Open*.

eMethods. Cohort Definition and Diagnosis and Procedure Codes

Section A: Cohort Definition

DEFINING THE COHORT

Our cohort includes beneficiaries with incident early stage or surgically managed colon cancer. Patients were identified using a modified Setoguchi algorithm presented by Bronson et al[21].

Incident cancers are defined as cancers first diagnosed within the study period (04/01/2016 - 09/30/2018) based on three identification rules using ICD-10 or CPT codes to identify diagnosis and type of cancer or cancer treatment (including biopsy, surgical procedures, radiation therapy, chemotherapy, and complications related to cancer). Prior to the start of the study period, there is a clean look-back period from 10/01/2015 to 03/31/3016 to identify and remove all prevalent cancers (**Figure A1**). Beneficiaries must be alive at the start of the study period (04/01/2016) to be included in the analyses. Outcomes will be studied through 09/30/2018.

Inclusion Criteria:
Per the algorithm, the following rules are used to identify an incident cancer (**Figure A1**):

1) At least one cancer ICD diagnosis code (**Table A1**) during a hospitalization or outpatient visit <u>and</u> a CPT code related to chemotherapy (**Table A2**), radiation therapy (**Table A3**), or cancer-directed surgery (**Table A4**).

   OR

2) At least two cancer ICD diagnosis codes (**Table A1**) on different dates within 12 months following a CPT code for cancer-directed biopsy (**Table A5**).

   OR

3) One cancer ICD diagnosis code (**Table A1**) within 14 days of a diagnosis or procedure code for a cancer-related symptoms (**Table A6**) <u>and</u> another within the 12 months following the symptom.

© 2022 Ramkumar N et al. *JAMA Network Open*.

Figure A1. Identifying incident colon cancers among Medicare beneficiaries.

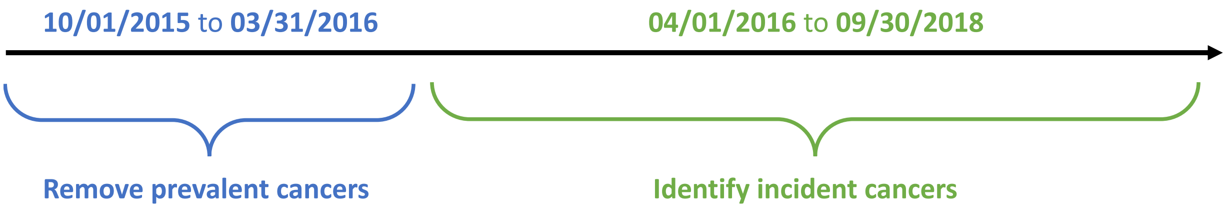

1. At least one cancer ICD diagnosis code (**Table A1**) during a hospitalization <u>or</u> outpatient visit with a CPT code related to chemotherapy (**Table A2**), radiation therapy (**Table A3**), or cancer-directed surgery (**Table A4**).

   OR

2. At least two cancer ICD diagnosis codes (**Table A1**) on different dates within 12 months following a CPT code for cancer-directed biopsy (**Table A5**).

   OR

3. One cancer ICD diagnosis code (**Table A1**) within 14 days of a CPT code for a cancer-related complication (**Table A6**) <u>and</u> another within the 12 months following the complication.

Exclusion Criteria:
We excluded beneficiaries with who met the following criteria:

Look-back period (10/1/2015-03/31/2016):

1) Nursing home residents from cohort by extracting carrier claims corresponding to nursing home HCPS/CPT codes (10/01/2015-12/31/2018)
    - Keep carrier records corresponding to first and last visits (minimum and maximum dates)
    - If the difference between max and min dates are >90 days, we consider them to be nursing home residents in that year
    - HCPCS_CD: 99301-99318
2) Under 65 years old
3) HMO enrollees and beneficiaries without full Part A and Part B enrollment
4) With end-stage renal disease (denominator file CCW for 2015)
5) Diagnosis of colon cancer (**Table A1**) or colon cancer-directed surgery (**Table A4**)
6) With HCC 8-12 (8: Metastatic cancer and acute leukemia; 9: lung and other severe cancers; 10: lymphoma and other cancers; 11: colorectal, bladder, and other cancers; 12: breast, prostate, and other cancers, and tumors)

Incident Cancer Period (04/01/2016-09/30/2018)

7) Metastatic cancer within 3 months of initial diagnosis, defined as 1 outpatient or inpatient visit with secondary metastatic ICD-10-CM diagnosis code (**Table B1)**.
8) Patients with rectal cancer. Look for most recent claim within 60 days of index diagnosis and compare frequency of rectal vs colon cancer diagnosis codes (**Table A1**); define using cancer with highest frequency of codes. If equal, call rectal cancer.

IMPUTING THE AREA DEPRIVATION INDEX

We imputed the ADI rank using the mean values of five iterations of the imputation model that included the following variables at the ZIP+4 level: proportion below federal poverty line, median household income, and percent with less than high school education.

© 2022 Ramkumar N et al. *JAMA Network Open*.

Table A1. ICD-10-CM diagnosis codes for colon and rectal cancer.

Code	Description	Side	Source
Colon Cancer			
**C18.0**	Malignant neoplasm of cecum	Right	[36]
**C18.2**	Malignant neoplasm of ascending colon	Right	[36]
**C18.3**	Malignant neoplasm of hepatic flexure	Right	[37]
**C18.4**	Malignant neoplasm of transverse colon	Right	[37]
**C18.5**	Malignant neoplasm of splenic flexure	Right	[36]
**C18.6**	Malignant neoplasm of descending colon	Left	[36]
**C18.7**	Malignant neoplasm of sigmoid colon	Left	[36]
**C18.8**	Malignant neoplasm of overlapping sites of colon	-	
**C18.9**	Malignant neoplasm of colon, unspecified	-	
**C19**	Malignant neoplasm of rectosigmoid junction	Left	[36]
Rectal Cancer			
**C20**	Malignant neoplasm of rectum	N/A	

Defining Side

To define right vs. left sided cancer, look +60 days beyond index diagnosis date to find most recent claim, and assign side according to the ICD-10-CM codes listed in that claim per **Table A1**. Combinations of codes should be categorized as follows:

- **Right** = Right only
  Right AND unspecified
  Right AND left
  Right AND left AND unspecified
- **Left** = Left only
  Left AND unspecified
- **Unspecified** = unspecified only

© 2022 Ramkumar N et al. *JAMA Network Open*.

Table A2. Codes for cancer-related chemotherapy. (Adapted from Bronson et al supplement and replicates lung cancer cohort with the addition of capecitabine HCPCS codes)

**Code**	**Description**	**Source**
CPT		
**36640**	Arterial catheterization for prolonged infusion therapy (chemotherapy), cutdown	[21]
**51720**	Bladder instillation of anticarcinogenic agent (including detention time)	[21]
**96401**	Under injection and intravenous infusion chemotherapy and other highly complex drug or highly complex biologic agent administration	[21]
**96402**	Chemotherapy administration, subcutaneous or intramuscular:hormonal anti-neoplastic	[21]
**96405**	Chemotherapy administration, intralesional; up to and including 7 lesions	[21]
**96406**	Chemotherapy administration, intralesional; more than 7 lesions	[21]
**96409**	Under injection and intravenous infusion chemotherapy and other highly complex drug or highly complex biologic agent administration	[21]
**96413**	Under injection and intravenous infusion chemotherapy and other highly complex drug or highly complex biologic agent administration	[21]
**96415**	Under injection and intravenous infusion chemotherapy and other highly complex drug or highly complex biologic agent administration	[21]
**96416**	Under injection and intravenous infusion chemotherapy and other highly complex drug or highly complex biologic agent administration	[21]
**96420**	Chemotherapy administration, intra-arterial; push technique	[21]
**96422**	Chemotherapy administration, intra-arterial; infusion technique, up to one hour	[21]
**96423**	Chemotherapy administration, intra-arterial; infusion technique, one to 8 hours, each additional hour (list separately in addition to code for	[21]
**96425**	Chemotherapy administration, intra-arterial; infusion technique, initiation of prolonged infusion (more than 8 hours), requiring the use of a p	[21]
**96440**	Chemotherapy administration into pleural cavity, requiring and including thoracentesis	[21]
**96446**	Under Other Injection and Infusion Services	[21]
**96450**	Chemotherapy administration, into cns (eg, intrathecal), requiring and including spinal puncture	[21]
**96521**	Refilling and maintenance of portable pump	[21]
**96522**	Refilling and maintenance of implantable pump or reservoir for drug delivery, systemic (eg, intravenous, intra-arterial)	[21]
**96542**	Chemotherapy injection, subarachnoid or intraventricular via subcutaneous reservoir, single or multiple agents	[21]
**96549**	Unlisted chemotherapy procedure	[21]
ICD-10 CM		
**Z51.11**	Encounter for antineoplastic chemotherapy	[21]
**Z51.12**	Encounter for antineoplastic immunotherapy	[21]
ICD-10-PCS		
**3E0.3305**	Introduction of Other Antineoplastic into Peripheral Vein, Percutaneous Approach	[21]
**3E0.4305**	Introduction of Other Antineoplastic into Central Vein, Percutaneous Approach	[21]

**XW0.3351**	Introduction of Blinatumomab Antineoplastic Immunotherapy into Peripheral Vein, Percutaneous Approach, New Technology Group 1	[21]
**XW0.33B3**	Introduction of Cytarabine and Daunorubicin Liposome Antineoplastic into Peripheral Vein, Percutaneous Approach, New Technology Group 3	[21]
**XW0.33C3**	Introduction of Engineered Autologous Chimeric Antigen Receptor T-cell Immunotherapy into Peripheral Vein, Percutaneous Approach, New Technology Group 3	[21]
**XW0.4351**	Introduction of Blinatumomab Antineoplastic Immunotherapy into Central Vein, Percutaneous Approach, New Technology Group 1	[21]
**XW0.43B3**	Introduction of Cytarabine and Daunorubicin Liposome Antineoplastic into Central Vein, Percutaneous Approach, New Technology Group 3	[21]
**XW0.43C3**	Introduction of Engineered Autologous Chimeric Antigen Receptor T-cell Immunotherapy into Central Vein, Percutaneous Approach, New Technology Group 3	[21]
HCPCS		
**Q0083***	Chemotherapy administration by other than infusion technique only (e.g., subcutaneous, intramuscular, push)	[21]
**Q0084***	Chemotherapy administration by infusion technique only, per visit	[21]
**Q0085***	Chemotherapy administration by both infusion technique and other technique(s) (e.g., subcutaneous, intramuscular, push), per visit	[21]
**J9000-J9999***	Chemotherapy agents	[21]
**J8520**	Capecitabine	
**J8521**	Capecitabine	
**WW089**	Capecitabine	
**WW090**	Capecitabine	
**WW091**	Capecitabine	
**WW093**	Capecitabine	
**WW094**	Capecitabine	
**WW096**	Capecitabine	
**C9474**	Irinotecan	
**Q5107**	Bevacizumab	
**Q5118**	Bevacizumab	
**C9025**	Ramucirumab	
**C9027**	Pembrolizumab	
**C9453**	Nivolumab	
Revenue Center		
**0331**		
**0332**		
**0335**		
NDC in DME file		
**000041100**	Capecitabine	
**000041101**	Capecitabine	
**000540271**	Capecitabine	
**000540272**	Capecitabine	
**000937473**	Capecitabine	
**000937474**	Capecitabine	

© 2022 Ramkumar N et al. *JAMA Network Open*.

**001790149**	Capecitabine	
**001790195**	Capecitabine	
**001790229**	Capecitabine	
**003782511**	Capecitabine	
**003782512**	Capecitabine	
**153380237**	Capecitabine	
**153380255**	Capecitabine	
**153380335**	Capecitabine	
**163640072**	Capecitabine	
**163640073**	Capecitabine	
**167140467**	Capecitabine	
**167140468**	Capecitabine	
**167290072**	Capecitabine	
**167290073**	Capecitabine	
**422910166**	Capecitabine	
**422910167**	Capecitabine	
**422910190**	Capecitabine	
**422910191**	Capecitabine	
**502680154**	Capecitabine	
**510790510**	Capecitabine	
**514070095**	Capecitabine	
**514070096**	Capecitabine	
**538080411**	Capecitabine	
**548684143**	Capecitabine	
**548685260**	Capecitabine	
**596510204**	Capecitabine	
**596510205**	Capecitabine	
**597650072**	Capecitabine	
**597650073**	Capecitabine	
**606870149**	Capecitabine	
**637593000**	Capecitabine	
**637593001**	Capecitabine	
**649800276**	Capecitabine	
**649800277**	Capecitabine	
**651620843**	Capecitabine	
**651620844**	Capecitabine	
**678770458**	Capecitabine	
**678770459**	Capecitabine	
**690970948**	Capecitabine	
**690970949**	Capecitabine	
**695390019**	Capecitabine	
**695390020**	Capecitabine	
**722050006**	Capecitabine	
**722050007**	Capecitabine	
**724850204**	Capecitabine	
**724850205**	Capecitabine	

NB: Including capecitabine because it is in an important drug for colon cancer captured in the Part B claims but not included in the J9000-J9999 range.

© 2022 Ramkumar N et al. *JAMA Network Open*.

Table A3. CPT Codes for cancer-related, non-surgical, radiation therapy. (Adapted from Bronson et al supplement with codes added on expert review by NK)

CPT	Description	Source
**55860**	Exposure of prostate, any approach, for insertion of radioactive substance;	[21]
**55862**	Exposure of prostate, any approach, for insertion of radioactive substance; with lymph node biopsy(s) (limited pelvic lymphadenectomy)	[21]
**55865**	Exposure of prostate, any approach, for insertion of radioactive substance; with bilateral pelvic lymphadenectomy, including external iliac	[21]
**55875**	Transperineal placement of needles or catheters into prostate for interstitial radioelement application, with or without cystoscopy	[21]
**77261**	Therapeutic radiology treatment planning; simple	[21]
**77262**	Therapeutic radiology treatment planning; intermediate	[21]
**77263**	Therapeutic radiology treatment planning; complex	[21]
**77280**	Therapeutic radiology simulation-aided field setting; simple	[21]
**77285**	Therapeutic radiology simulation-aided field setting; intermediate	[21]
**77290**	Therapeutic radiology simulation-aided field setting; complex	[21]
**77295**	Therapeutic radiology simulation-aided field setting; by three-dimensional reconstruction of tumor volume	[21]
**77299**	Unlisted procedure, therapeutic radiology clinical treatment planning	[21]
**77300**	Basic rad dosimetry calc, centrl axis dpth dos calc,tdf,nsd,gap calc,off axis factor,tiss inhomogeneity factors, calc,non-ionizing rad surf&depth	[21]
**77301**	IMRT dose planning	**NK**
**77306**	Simple teletherapy isodose plan when there are one or two unmodified ports directly at one volume of interest.	[21]
**77307**	Complex teletherapy isodose plan when multiple treatment areas, tangential ports, the use of wedges, blocking, rotational beams, or special beam considerations will be used.	[21]
**77316**	Brachytherapy isodose plan	[21]
**77317**	Brachytherapy isodose plan	[21]
**77318**	Brachytherapy isodose plan	[21]
**77321**	Special teletherapy port plan, particles, hemi-body, total body	[21]
**77331**	Special dosimetry (eg, tld, microdosimetry) (specify), only when prescribed by the treating physician	[21]
**77332**	Treatment devices, design and construction; simple (simple block, simple bolus)	[21]
**77333**	Treatment devices, design and construction; intermediate (multiple blocks, stents, bite blocks, special bolus)	[21]
**77334**	Treatment devices, design and construction; complex (irregular blocks, special shields, compensators, wedges, molds or casts)	[21]
**77336**	Continuing medical physics consult, incl assessment of treat parameters, quality assurance of dose delivery, & review of patient treat doc in s	[21]
**77338**	MLC device for IMRT	**NK**
**77370**	Special medical radiation physics consultation	[21]
**77372**	SRS linear based	
**77373**	SBRI, 1 or more lesions, 1-5 fractions	
**77385**	IMRT treatment delivery, includes guidance and tracking when performed, simple	
**77386**	IMRT treatment delivery, includes guidance and tracking when performed, complex	

© 2022 Ramkumar N et al. *JAMA Network Open*.

**77399**	Unlisted procedure, medical radiation physics, dosimetry and treatment devices, and special services	[21]
**77401**	Radiation treatment delivery, superficial and/or ortho voltage	[21]
**77402**	Radiation treatment delivery, single treatment area, single port or parallel opposed ports, simple blocks or no blocks; up to 5 mev	[21]
**77407**	Radiation treatment delivery, two separate treatment areas, three or more ports on a single treatment area, use of multiple blocks; up to 5 mev	[21]
**77412**	Radiation treatment delivery, >=3 separate treatment areas, custom blocking, tangential ports, wedges, rotational beam, compensators, spec part	[21]
**77417**	Therapeutic radiology port film(s)	[21]
**77424**	IORT delivery, x-ray, single treatment session	
**77425**	Intraoperative radiation treatment delivery, electrons, single treatment session	[21]
**77431**	Radiation therapy management with complete course of therapy consisting of one or two fractions only	[21]
**77432**	Stereotactic radiation treatment management of cerebral lesion(s) (complete course of treatment consisting of one session)	[21]
**77470**	Special treatment procedure (eg, total body irradiation, hemibody radiation, per oral, endocavitary or intraoperative cone irradiation)	[21]
**77499**	Unlisted procedure, therapeutic radiology treatment management	[21]
**77520**	Proton treatment, simple, without compensation	
**77522**	Proton treatment, simple, with compensation	
**77523**	Proton treatment, intermediate	
**77525**	Proton treatment, complex	
**77750**	Infusion or instillation of radioelement solution	[21]
**77761**	Intracavitary radiation source application; simple	[21]
**77762**	Intracavitary radiation source application; intermediate	[21]
**77763**	Intracavitary radiation source application; complex	[21]
**77770**	Clinical brachytherapy radiation treatment	[21]
**77771**	Clinical brachytherapy radiation treatment	[21]
**77772**	Clinical brachytherapy radiation treatment	[21]
**77778**	Interstitial radiation source application; complex	[21]
**77789**	Surface application of radiation source	[21]
**77790**	Supervision, handling, loading of radiation source	[21]
**77799**	Unlisted procedure, clinical brachytherapy	[21]
**79200**	Intracavitary radioactive colloid therapy	[21]
**79300**	Interstitial radioactive colloid therapy	[21]
**79440**	Intra-articular radiopharmaceutical therapy	[21]
**79999**	Unlisted radiopharmaceutical therapeutic procedure	[21]
**G6003**	Single treatment area, single port or parallel opposed ports, simple blocks or no blocks, up to SMeV	
**G6004**	Single treatment area, single port or parallel opposed ports, simple blocks or no blocks, 6-10MeV	
**G6005**	Single treatment area, single port or parallel opposed ports, simple blocks or no blocks, 11-19MeV	
**G6006**	Single treatment area, single port or parallel opposed ports, simple blocks or no blocks, 20MeV or greater	
**G6007**	2 separate treatment areas, 3 or more ports on a single treatment area, use of multiple blocks, up to SMeV	
**G6008**	2 separate treatment areas, 3 or more ports on a single treatment area, use of multiple blocks, 6-10MeV	

© 2022 Ramkumar N et al. *JAMA Network Open*.

**G6009**	2 separate treatment areas, 3 or more ports on a single treatment area, use of multiple blocks, 11-19MeV	
**G6010**	2 separate treatment areas, 3 or more ports on a single treatment area, use of multiple blocks, 20MeV or greater	
**G6011**	3 or more separate treatment areas, custom blocking, tangential ports, wedges, rotational beam, compensators, electron beam, up to SMeV	
**G6012**	3 or more separate treatment areas, custom blocking, tangential ports, wedges, rotational beam, compensators, electron beam, 6-10MeV	
**G6013**	3 or more separate treatment areas, custom blocking, tangential ports, wedges, rotational beam, compensators, electron beam, 11-19MeV	
**G6014**	3 or more separate treatment areas, custom blocking, tangential ports, wedges, rotational beam, compensators, electron beam, 20MeV or greater	
**G6015**	IMRT treatment delivery	
**G6016**	Compensator-based IMRT delivery	

© 2022 Ramkumar N et al. *JAMA Network Open*.

Table A4. CPT codes for cancer-related, non-surgical biopsy. (Adapted from Bronson et al supplement and replicates lung cancer cohort).

Code	Description	Source
**44100**	Biopsy of intestine by capsule, tube, peroral (one or more specimens)	21
**45305**	Proctosigmoidoscopy, rigid; with biopsy, single or multiple	21
**45308**	Proctosigmoidoscopy, rigid; with removal of single tumor, polyp, or other lesion by hot biopsy forceps or bipolar cautery	21
**45309**	Proctosigmoidoscopy, rigid; with removal of single tumor, polyp, or other lesion by snare technique	21
**45315**	Proctosigmoidoscopy, rigid; with removal of multiple tumors, polyps, or other lesions by hot biopsy forceps, bipolar cautery or snare technique	21
**45317**	Proctosigmoidoscopy, rigid; with control of bleeding (eg, injection, bipolar cautery, unipolar cautery, laser, heater probe, stapler, plasma co	21
**45320**	Proctosigmoidoscopy, rigid; with ablation of tumor(s), polyp(s), or other lesion(s) not amenable to removal by hot biopsy forceps, bipolar caut	21
**45331**	Sigmoidoscopy, flexible; with biopsy, single or multiple	21
**45333**	Sigmoidoscopy, flexible; with removal of tumor(s), polyp(s), or other lesion(s) by hot biopsy forceps or bipolar cautery	21
**45338**	Sigmoidoscopy, flexible; with removal of tumor(s), polyp(s), or other lesion(s) by snare technique	21
**45339**	Sigmoidoscopy, flexible; with ablation of tumor(s), polyp(s), or other lesion(s) not amenable to removal by hot biopsy forceps, bipolar cautery	21
**45341**	Sigmoidoscopy, flexible; with endoscopic ultrasound examination	21
**45342**	Sigmoidoscopy, flexible; with transendoscopic ultrasound guided intramural or transmural fine needle aspiration/biopsy(s)	21
**45355**	Colonoscopy, rigid or flexible, transabdominal via colotomy, single or multiple	21
**45380**	Colonoscopy, flexible, proximal to splenic flexure; with biopsy, single or multiple	21
**45383**	Colonoscopy, flexible, proximal to splenic flexure; with ablation of tumor(s), polyp(s), or other lesion(s) not amenable to removal by hot biop	21
**45384**	Colonoscopy, flexible, proximal to splenic flexure; with removal of tumor(s), polyp(s), or other lesion(s) by hot biopsy forceps or bipolar cau	21
**45385**	Colonoscopy, flexible, proximal to splenic flexure; with removal of tumor(s), polyp(s), or other lesion(s) by snare technique	21
**88305**	Level IV - Surgical pathology, gross and microscopic examination is for the gross and microscopic examination of a specimen to provide a diagnosis	21
**88309**	Level VI - surgical path, gross & micro exam bone resection breast, mastectomy - with regional lymph nodes colon, segmental resection for tumor	21

© 2022 Ramkumar N et al. *JAMA Network Open*.

Table A5. Codes for cancer-directed surgical treatments. Note, the codes listed under the laparoscopic procedure codes in blue are the complementary open procedure code. (Adapted from Bronson et al supplement and literature).

Code	Description	Source
CPT		
**44140**	Colectomy, partial; with anastomosis	21,5
**44141**	Colectomy, partial; with skin level cecostomy or colostomy	21,5
**44143**	Colectomy, partial; with end colostomy and closure of distal segment (hartmann type procedure)	21,5
**44144**	Colectomy, partial; with resection, with colostomy or ileostomy and creation of mucofistula	21,5
**44145**	Colectomy, partial; with coloproctostomy (low pelvic anastomosis)	21,5
**44146**	Colectomy, partial; with coloproctostomy (low pelvic anastomosis), with colostomy	21,5
**44147**	Colectomy, partial; abdominal and transanal approach	21,5
**44150**	Colectomy, total, abdominal, without proctectomy; with ileostomy or ileoproctostomy	21,5
**44151**	Colectomy, total, abdominal, without proctectomy; with continent ileostomy	21,5
**44152**	Colectomy, total, abdominal, without proctectomy; with rectal mucosectomy, ileoanal anastomosis, with or without loop ileostomy	21,5
**44153**	Colectomy, total, abdominal, without proctectomy; with rectal mucosectomy, ileoanal anastomosis, creation of ileal reservoir (s or j), with or without loop ileostomy	21,5
**44155**	Colectomy, total, abdominal, with proctectomy; with ileostomy	21,5
**44156**	Colectomy, total, abdominal, with proctectomy; with continent ileostomy	21,5
**44157**	Colectomy, total; abdominal, without proctectomy; with ileoanal anastomosis, includes loop ileostomy, and rectal mucosectomy, when performed	26
**44158**	Colectomy, total, abdominal, without proctectomy, with ileoanal anastomosis, creation of ileal reservoir (s or j), includes loop ileostomy, and rectal mucosectomy, when performed	26
**44160**	Colectomy, partial, with removal of terminal ileum with ileocolostomy	21,5
**44204** (44140)	Laparoscopy, surgical; colectomy, partial, with anastomosis	21,5
**44205** (44160)	Laparoscopy, surgical; colectomy, partial, with removal of terminal ileum with ileocolostomy	21,5
**44206** (44143)	Laparoscopy, surgical; colectomy, partial, with end colostomy and closure of distal segment (hartmann type procedure)	21,5
**44207** (44145)	Laparoscopy, surgical; colectomy, partial, with anastomosis, with coloproctostomy (low pelvic anastomosis)	21,5
**44208** (44146)	Laparoscopy, surgical; colectomy, partial, with anastomosis, with coloproctostomy (low pelvic anastomosis) with colostomy	21,5
**44210** (44150)	Laparoscopy, surgical; colectomy, total, abdominal, without proctectomy, with ileostomy or ileoproctostomy	21,5
**44211** (44158)	Laparoscopy, surgical; colectomy, total, abdominal, with proctectomy, with ileoanal anastomosis, creation of ileal reservoir (s or j), with loop ileostomy, includes rectal mucosectomy, when performed	21,5
**44212** (44155)	Laparoscopy, surgical; colectomy, total, abdominal, with proctectomy, with ileostomy	21,5

© 2022 Ramkumar N et al. *JAMA Network Open*.

ICD-10-PCS		
**0DTE4ZZ**	Resection of Large Intestine, Percutaneous Endoscopic Approach	[38]
**0DTF4ZZ**	Resection of Right Large Intestine, Percutaneous Endoscopic Approach	[38]
**0DTG4ZZ**	Resection of Left Large Intestine, Percutaneous Endoscopic Approach	[38]
**0DTH4ZZ**	Resection of Cecum, Percutaneous Endoscopic Approach	[38]
**0DTK4ZZ**	Resection of Ascending Colon, Percutaneous Endoscopic Approach	[38]
**0DTL4ZZ**	Resection of Transverse Colon, Percutaneous Endoscopic Approach	[38]
**0DTM4ZZ**	Resection of Descending Colon, Percutaneous Endoscopic Approach	[38]
**0DTN4ZZ**	Resection of Sigmoid Colon, Percutaneous Endoscopic Approach	[38]
**0DTE8ZZ**	Resection of Large Intestine, Via Natural or Artificial Opening Endoscopic	[38]
**0DTF8ZZ**	Resection of Right Large Intestine, Via Natural or Artificial Opening Endoscopic	[38]
**0DTG8ZZ**	Resection of Left Large Intestine, Via Natural or Artificial Opening Endoscopic	[38]
**0DTH8ZZ**	Resection of Cecum, Via Natural or Artificial Opening Endoscopic	[38]
**0DTK8ZZ**	Resection of Ascending Colon, Via Natural or Artificial Opening Endoscopic	[38]
**0DTL8ZZ**	Resection of Transverse Colon, Via Natural or Artificial Opening Endoscopic	[38]
**0DTM8ZZ**	Resection of Descending Colon, Via Natural or Artificial Opening Endoscopic	[38]
**0DTN8ZZ**	Resection of Sigmoid Colon, Via Natural or Artificial Opening Endoscopic	[38]
**0DTEFZZ**	Resection of Large Intestine, Open Approach	[38]
**0DTFFZZ**	Resection of Right Large Intestine, Via Natural or Artificial Opening	[38]
**0DTGFZZ**	Resection of Left Large Intestine, Via Natural or Artificial Opening	[38]
**0DTLFZZ**	Resection of Transverse Colon, Via Natural or Artificial Opening With Percutaneous Endoscopic Assistance	[38]
**0DTMFZZ**	Resection of Descending Colon, Via Natural or Artificial Opening With Percutaneous Endoscopic Assistance	[38]
**0DTNFZZ**	Resection of Sigmoid Colon, Via Natural or Artificial Opening With Percutaneous Endoscopic Assistance	[38]
**0DTE0ZZ**	Resection of Large Intestine, Open Approach	[38]
**0DTF0ZZ**	Resection of Right Large Intestine, Open Approach	[38]
**0DTG0ZZ**	Resection of Left Large Intestine, Open Approach	[38]
**0DTH0ZZ**	Resection of Cecum, Open Approach	[38]
**0DTK0ZZ**	Resection of Ascending Colon, Open Approach	[38]
**0DTL0ZZ**	Resection of Transverse Colon, Open Approach	[38]
**0DTM0ZZ**	Resection of Descending Colon, Open Approach	[38]
**0DTN0ZZ**	Resection of Sigmoid Colon, Open Approach	[38]
**0DTE7ZZ**	Resection of Large Intestine, Via Natural or Artificial Opening	[38]
**0DTF7ZZ**	Resection of Right Large Intestine, Via Natural or Artificial Opening	[38]
**0DTG7ZZ**	Resection of Left Large Intestine, Via Natural or Artificial Opening	[38]
**0DTH7ZZ**	Resection of Cecum, Via Natural or Artificial Opening	[38]
**0DTK7ZZ**	Resection of Ascending Colon, Via Natural or Artificial Opening	[38]
**0DTL7ZZ**	Resection of Transverse Colon, Via Natural or Artificial Opening	[38]
**0DTM7ZZ**	Resection of Descending Colon, Via Natural or Artificial Opening	[38]
**0DTN7ZZ**	Resection of Sigmoid Colon, Via Natural or Artificial Opening	[38]
**8E0W0CZ**	Robotic Assisted Procedure of Trunk Region, Open Approach	[38,39]
**8E0W3CZ**	Robotic Assisted Procedure of Trunk Region, Percutaneous Approach	[38,39]
**8E0W4CZ**	Robotic Assisted Procedure of Trunk Region, Percutaneous Endoscopic Approach	[38,39]
**8E0W7CZ**	Robotic Assisted Procedure of Trunk Region, Via Natural or Artificial Opening	[38,39]

© 2022 Ramkumar N et al. *JAMA Network Open*.

**8E0W8CZ**	Robotic Assisted Procedure of Trunk Region, Via Natural or Artificial Opening Endoscopic	[38,39]

© 2022 Ramkumar N et al. *JAMA Network Open*.

Table A6. ICD-10-CM diagnosis codes for cancer-related symptoms used to identify incident cancer. (Replicated from Bronson[21] et al supplement)

Code	Description	Source
**E83.50**	Unspecified disorder of calcium metabolism	21
**E83.52**	Hypercalcemia	21
**E20.1**	Pseudohypoparathyroidism	21
**E83.59**	Other disorders of calcium metabolism	21
**C79.31**	Secondary malignant neoplasm of brain	21
**G95.9**	Disease of spinal cord, unspecified	21
**K56.5**	Intestinal adhesions [bands] with obstruction (postprocedural) (postinfection)	21
**K56.69**	Other intestinal obstruction	21
**K56.60**	Unspecified intestinal obstruction	21

© 2022 Ramkumar N et al. *JAMA Network Open*.

Section B: Code List for Exclusion Criteria

Table B1. ICD-10-CM codes for secondary neoplasms to indicate metastatic disease. NB: list does not include C77.2 (intra-abdominal nodes)

Code	Description	Source
**C77.0**	Sec and unsp malig neoplasm of nodes of head, face and neck	
**C77.1**	Secondary and unsp malignant neoplasm of intrathorac nodes	
**C77.3**	Sec and unsp malig neoplasm of axilla and upper limb nodes	
**C77.4**	Sec and unsp malig neoplasm of inguinal and lower limb nodes	
**C77.5**	Secondary and unsp malignant neoplasm of intrapelv nodes	
**C77.8**	Sec and unsp malig neoplasm of nodes of multiple regions	
**C78.00**	Secondary malignant neoplasm of unspecified lung	
**C78.01**	Secondary malignant neoplasm of right lung	
**C78.02**	Secondary malignant neoplasm of left lung	
**C78.1**	Secondary malignant neoplasm of mediastinum	
**C78.2**	Secondary malignant neoplasm of pleura	
**C78.30**	Secondary malignant neoplasm of unsp respiratory organ	
**C78.39**	Secondary malignant neoplasm of other respiratory organs	
**C78.4**	Secondary malignant neoplasm of small intestine	
**C78.6**	Secondary malignant neoplasm of retroperiton and peritoneum	
**C78.7**	Secondary malig neoplasm of liver and intrahepatic bile duct	
**C78.80**	Secondary malignant neoplasm of unspecified digestive organ	
**C78.89**	Secondary malignant neoplasm of other digestive organs	
**C79.00**	Secondary malignant neoplasm of unsp kidney and renal pelvis	
**C79.01**	Secondary malignant neoplasm of r kidney and renal pelvis	
**C79.02**	Secondary malignant neoplasm of left kidney and renal pelvis	
**C79.10**	Secondary malignant neoplasm of unspecified urinary organs	
**C79.11**	Secondary malignant neoplasm of bladder	
**C79.19**	Secondary malignant neoplasm of other urinary organs	
**C79.2**	Secondary malignant neoplasm of skin	
**C79.31**	Secondary malignant neoplasm of brain	
**C79.32**	Secondary malignant neoplasm of cerebral meninges	
**C79.40**	Secondary malignant neoplasm of unsp part of nervous system	
**C79.49**	Secondary malignant neoplasm of oth parts of nervous system	
**C79.51**	Secondary malignant neoplasm of bone	
**C79.52**	Secondary malignant neoplasm of bone marrow	
**C79.60**	Secondary malignant neoplasm of unspecified ovary	
**C79.61**	Secondary malignant neoplasm of right ovary	
**C79.62**	Secondary malignant neoplasm of left ovary	
**C79.70**	Secondary malignant neoplasm of unspecified adrenal gland	
**C79.71**	Secondary malignant neoplasm of right adrenal gland	
**C79.72**	Secondary malignant neoplasm of left adrenal gland	
**C79.81**	Secondary malignant neoplasm of breast	
**C79.82**	Secondary malignant neoplasm of genital organs	
**C79.89**	Secondary malignant neoplasm of other specified sites	
**C79.9**	Secondary malignant neoplasm of unspecified site	

© 2022 Ramkumar N et al. *JAMA Network Open*.

Section C: Code List for Outcomes

Table C1. Codes to identify cancer-directed surgery. Note, the codes listed under the laparoscopic procedure codes in blue are the complementary open procedure code. (Adapted from Bronson et al supplement and literature search).

Code	Description	Source
CPT		
**44140**	Colectomy, partial; with anastomosis	21,5
**44141**	Colectomy, partial; with skin level cecostomy or colostomy	21,5
**44143**	Colectomy, partial; with end colostomy and closure of distal segment (hartmann type procedure)	21,5
**44144**	Colectomy, partial; with resection, with colostomy or ileostomy and creation of mucofistula	21,5
**44145**	Colectomy, partial; with coloproctostomy (low pelvic anastomosis)	21,5
**44146**	Colectomy, partial; with coloproctostomy (low pelvic anastomosis), with colostomy	21,5
**44147**	Colectomy, partial; abdominal and transanal approach	21,5
**44150**	Colectomy, total, abdominal, without proctectomy; with ileostomy or ileoproctostomy	21,5
**44151**	Colectomy, total, abdominal, without proctectomy; with continent ileostomy	21,5
**44152**	Colectomy, total, abdominal, without proctectomy; with rectal mucosectomy, ileoanal anastomosis, with or without loop ileostomy	21,5
**44153**	Colectomy, total, abdominal, without proctectomy; with rectal mucosectomy, ileoanal anastomosis, creation of ileal reservoir (s or j), with or without loop ileostomy	21,5
**44155**	Colectomy, total, abdominal, with proctectomy; with ileostomy	21,5
**44156**	Colectomy, total, abdominal, with proctectomy; with continent ileostomy	21,5
**44157**	Colectomy, total; abdominal, without proctectomy; with ileoanal anastomosis, includes loop ileostomy, and rectal mucosectomy, when performed	26
**44158**	Colectomy, total, abdominal, without proctectomy, with ileoanal anastomosis, creation of ileal reservoir (s or j), includes loop ileostomy, and rectal mucosectomy, when performed	26
**44160**	Colectomy, partial, with removal of terminal ileum with ileocolostomy	21,5
**44204** (44140)	Laparoscopy, surgical; colectomy, partial, with anastomosis	21,5
**44205** (44160)	Laparoscopy, surgical; colectomy, partial, with removal of terminal ileum with ileocolostomy	21,5
**44206** (44143)	Laparoscopy, surgical; colectomy, partial, with end colostomy and closure of distal segment (hartmann type procedure)	21,5
**44207** (44145)	Laparoscopy, surgical; colectomy, partial, with anastomosis, with coloproctostomy (low pelvic anastomosis)	21,5
**44208** (44146)	Laparoscopy, surgical; colectomy, partial, with anastomosis, with coloproctostomy (low pelvic anastomosis) with colostomy	21,5
**44210** (44150)	Laparoscopy, surgical; colectomy, total, abdominal, without proctectomy, with ileostomy or ileoproctostomy	21,5

© 2022 Ramkumar N et al. *JAMA Network Open*.

**44211** (44158)	Laparoscopy, surgical; colectomy, total, abdominal, with proctectomy, with ileoanal anastomosis, creation of ileal reservoir (s or j), with loop ileostomy, includes rectal mucosectomy, when performed	21,5
**44212** (44155)	Laparoscopy, surgical; colectomy, total, abdominal, with proctectomy, with ileostomy	21,5
ICD-10-PCS		
**0DTE4ZZ**	Resection of Large Intestine, Percutaneous Endoscopic Approach	38
**0DTF4ZZ**	Resection of Right Large Intestine, Percutaneous Endoscopic Approach	38
**0DTG4ZZ**	Resection of Left Large Intestine, Percutaneous Endoscopic Approach	38
**0DTH4ZZ**	Resection of Cecum, Percutaneous Endoscopic Approach	38
**0DTK4ZZ**	Resection of Ascending Colon, Percutaneous Endoscopic Approach	38
**0DTL4ZZ**	Resection of Transverse Colon, Percutaneous Endoscopic Approach	38
**0DTM4ZZ**	Resection of Descending Colon, Percutaneous Endoscopic Approach	38
**0DTN4ZZ**	Resection of Sigmoid Colon, Percutaneous Endoscopic Approach	38
**0DTE8ZZ**	Resection of Large Intestine, Via Natural or Artificial Opening Endoscopic	38
**0DTF8ZZ**	Resection of Right Large Intestine, Via Natural or Artificial Opening Endoscopic	38
**0DTG8ZZ**	Resection of Left Large Intestine, Via Natural or Artificial Opening Endoscopic	38
**0DTH8ZZ**	Resection of Cecum, Via Natural or Artificial Opening Endoscopic	38
**0DTK8ZZ**	Resection of Ascending Colon, Via Natural or Artificial Opening Endoscopic	38
**0DTL8ZZ**	Resection of Transverse Colon, Via Natural or Artificial Opening Endoscopic	38
**0DTM8ZZ**	Resection of Descending Colon, Via Natural or Artificial Opening Endoscopic	38
**0DTN8ZZ**	Resection of Sigmoid Colon, Via Natural or Artificial Opening Endoscopic	38
**0DTEFZZ**	Resection of Large Intestine, Open Approach	38
**0DTFFZZ**	Resection of Right Large Intestine, Via Natural or Artificial Opening	38
**0DTGFZZ**	Resection of Left Large Intestine, Via Natural or Artificial Opening	38
**0DTLFZZ**	Resection of Transverse Colon, Via Natural or Artificial Opening With Percutaneous Endoscopic Assistance	38
**0DTMFZZ**	Resection of Descending Colon, Via Natural or Artificial Opening With Percutaneous Endoscopic Assistance	38
**0DTNFZZ**	Resection of Sigmoid Colon, Via Natural or Artificial Opening With Percutaneous Endoscopic Assistance	38
**0DTE0ZZ**	Resection of Large Intestine, Open Approach	38
**0DTF0ZZ**	Resection of Right Large Intestine, Open Approach	38
**0DTG0ZZ**	Resection of Left Large Intestine, Open Approach	38
**0DTH0ZZ**	Resection of Cecum, Open Approach	38
**0DTK0ZZ**	Resection of Ascending Colon, Open Approach	38
**0DTL0ZZ**	Resection of Transverse Colon, Open Approach	38
**0DTM0ZZ**	Resection of Descending Colon, Open Approach	38
**0DTN0ZZ**	Resection of Sigmoid Colon, Open Approach	38
**0DTE7ZZ**	Resection of Large Intestine, Via Natural or Artificial Opening	38
**0DTF7ZZ**	Resection of Right Large Intestine, Via Natural or Artificial Opening	38
**0DTG7ZZ**	Resection of Left Large Intestine, Via Natural or Artificial Opening	38
**0DTH7ZZ**	Resection of Cecum, Via Natural or Artificial Opening	38
**0DTK7ZZ**	Resection of Ascending Colon, Via Natural or Artificial Opening	38
**0DTL7ZZ**	Resection of Transverse Colon, Via Natural or Artificial Opening	38
**0DTM7ZZ**	Resection of Descending Colon, Via Natural or Artificial Opening	38
**0DTN7ZZ**	Resection of Sigmoid Colon, Via Natural or Artificial Opening	38

© 2022 Ramkumar N et al. *JAMA Network Open*.

**8E0W0CZ**	Robotic Assisted Procedure of Trunk Region, Open Approach	[38,39]
**8E0W3CZ**	Robotic Assisted Procedure of Trunk Region, Percutaneous Approach	[38,39]
**8E0W4CZ**	Robotic Assisted Procedure of Trunk Region, Percutaneous Endoscopic Approach	[38,39]
**8E0W7CZ**	Robotic Assisted Procedure of Trunk Region, Via Natural or Artificial Opening	[38,39]
**8E0W8CZ**	Robotic Assisted Procedure of Trunk Region, Via Natural or Artificial Opening Endoscopic	[38,39]

© 2022 Ramkumar N et al. *JAMA Network Open*.

Table C2. Codes to identify colon cancer-directed chemotherapy.

HCPCS Code	Description	Source
Adjuvant-type drugs (chemotherapy agents that are appropriate for adjuvant therapy)		
**J9190**	5-fluorouracil (IV)	
**J9263**	Oxaliplatin (IV)	
**J8520**	Capecitabine	
**J8521**		
**WW089**		
**WW090**		
**WW091**		
**WW093**		
**WW094**		
**WW096**		
**000041100**	Capecitabine	
**000041101**	Capecitabine	
**000540271**	Capecitabine	
**000540272**	Capecitabine	
**000937473**	Capecitabine	
**000937474**	Capecitabine	
**001790149**	Capecitabine	
**001790195**	Capecitabine	
**001790229**	Capecitabine	
**003782511**	Capecitabine	
**003782512**	Capecitabine	
**153380237**	Capecitabine	
**153380255**	Capecitabine	
**153380335**	Capecitabine	
**163640072**	Capecitabine	
**163640073**	Capecitabine	
**167140467**	Capecitabine	
**167140468**	Capecitabine	
**167290072**	Capecitabine	
**167290073**	Capecitabine	
**422910166**	Capecitabine	
**422910167**	Capecitabine	
**422910190**	Capecitabine	
**422910191**	Capecitabine	
**502680154**	Capecitabine	
**510790510**	Capecitabine	
**514070095**	Capecitabine	
**514070096**	Capecitabine	
**538080411**	Capecitabine	
**548684143**	Capecitabine	
**548685260**	Capecitabine	
**596510204**	Capecitabine	
**596510205**	Capecitabine	
**597650072**	Capecitabine	

© 2022 Ramkumar N et al. *JAMA Network Open*.

**597650073**	Capecitabine	
**606870149**	Capecitabine	
**637593000**	Capecitabine	
**637593001**	Capecitabine	
**649800276**	Capecitabine	
**649800277**	Capecitabine	
**651620843**	Capecitabine	
**651620844**	Capecitabine	
**678770458**	Capecitabine	
**678770459**	Capecitabine	
**690970948**	Capecitabine	
**690970949**	Capecitabine	
**695390019**	Capecitabine	
**695390020**	Capecitabine	
**722050006**	Capecitabine	
**722050007**	Capecitabine	
**724850204**	Capecitabine	
**724850205**	Capecitabine	
Advanced colon cancer drug		
**C9474**	Irinotecan	
**J9205**		
**J9206**		
**J9035**	Bevacizumab	
**Q5107**		
**Q5118**		
**C9025**	Ramucirumab	
**J9308**		
**J9400**	Ziv-aflibercept	
**J9303**	Panitumumab	
**J9055**	Cetuximab	
**C9027**	Pembrolizumab	
**J9271**		
**C9453**	Nivolumab	
**J9299**		
**J9228**	Ipilimumab	

© 2022 Ramkumar N et al. *JAMA Network Open*.

Table C3. Codes to identify elective vs. non-elective cancer-directed surgeries.

To define non-elective cancer-directed surgeries, identify claims with a cancer-directed surgery (**Table C1**) and emergency room visits using the TDI algorithm below. Visits are identified in each data source using a unique date of service. If the same claim record was found in both files, only one record was sufficient to flag the claim as an emergency department visit.

Medicare File	Codes
Outpatient files	Revenue Center Codes 0450,0451,0452,0456,0459, 0981
MedPAR	Emergency Room Charge Amount > $0

© 2022 Ramkumar N et al. *JAMA Network Open*.

Table C4. Codes to identify minimally invasive (MIS, laparoscopic) versus open cancer-directed surgical intervention. (Adapted from Bronson et al supplement and literature search).

Open	MIS	Description	Source
CPT			
**44140**	**44204**	Colectomy, partial, with anastomosis	[21,5]
**44160**	**44205**	Colectomy, partial, with removal of terminal ileum with ileocolostomy	[21,5]
**44143**	**44206**	Colectomy, partial, with end colostomy and closure of distal segment (Hartmann type procedure)	[21,5]
**44145**	**44207**	Colectomy, partial, with anastomosis, with coloproctostomy (low pelvic anastomosis)	[21,5]
**44146**	**44208**	Colectomy, partial, with anastomosis, with coloproctostomy (low pelvic anastomosis) with colostomy	[21,5]
**44150**	**44210**	Colectomy, total, abdominal, without proctectomy, with ileostomy or ileoproctostomy	[21,5]
**44158**	**44211**	Colectomy, total, abdominal, with proctectomy, with ileoanal anastomosis, creation of ileal reservoir (s or j), with loop ileostomy, includes rectal mucosectomy, when performed	[21,5]
**44155**	**44212**	Colectomy, total, abdominal, with proctectomy, with ileostomy	[21,5]
**44141**	-	Colectomy, partial; with skin level cecostomy or colostomy	[21,5]
**44144**	-	Colectomy, partial; with resection, with colostomy or ileostomy and creation of mucofistula	[21,5]
**44147**	-	Colectomy, partial; abdominal and transanal approach	[21,5]
**44151**	-	Colectomy, total, abdominal, without proctectomy; with continent ileostomy	[21,5]
**44152**	-	Colectomy, total, abdominal, without proctectomy; with rectal mucosectomy, ileoanal anastomosis, with or without loop ileostomy	[21,5]
**44153**	-	Colectomy, total, abdominal, without proctectomy; with rectal mucosectomy, ileoanal anastomosis, creation of ileal reservoir (s or j), with or without loop ileostomy	[21,5]
**44156**	-	Colectomy, total, abdominal, with proctectomy; with ileostomy	[21,5]
**44157**	-	Colectomy, total, abdominal, with proctectomy; with continent ileostomy	[21,5]
ICD-10-PCS			
**0DTE0ZZ**	**0DTE4ZZ**	Resection of Large Intestine	[38]
**0DTE7ZZ**	**0DTE8ZZ**		[38]
	**0DTEFZZ**		[38]
**0DTF0ZZ**	**0DTF4ZZ**	Resection of Right Large Intestine	[38]
**0DTF7ZZ**	**0DTF8ZZ**		[38]
	**0DTFFZZ**		[38]
**0DTG0ZZ**	**0DTG4ZZ**	Resection of Left Large Intestine	[38]
**0DTG7ZZ**	**0DTG8ZZ**		[38]
	**0DTGFZZ**		[38]
**0DTH0ZZ**	**0DTH4ZZ**	Resection of Cecum	[38]
**0DTH7ZZ**	**0DTH8ZZ**		[38]
**0DTK0ZZ**	**0DTK4ZZ**	Resection of Ascending Colon	[38]
**0DTK7ZZ**	**0DTK8ZZ**		[38]

© 2022 Ramkumar N et al. *JAMA Network Open*.

**0DTL0ZZ**	**0DTL4ZZ**	Resection of Transverse Colon	[38]
**0DTL7ZZ**	**0DTK8ZZ**		[38]
	**0DTKFZZ**		[38]
**0DTM0ZZ**	**0DTM4ZZ**	Resection of Descending Colon	[38]
**0DTM7ZZ**	**0DTM8ZZ**		[38]
	**0DTMFZZ**		[38]
**0DTN0ZZ**	**0DTN4ZZ**	Resection of Sigmoid Colon	[38]
**0DTN7ZZ**	**0DTN8ZZ**		[38]
	**0DNFZZ**		[38]
	**8E0W0CZ**	Robotic Assisted Procedure of Trunk Region, Open Approach	[38]
	**8E0W3CZ**	Robotic Assisted Procedure of Trunk Region, Percutaneous Approach	[38]
	**8E0W4CZ**	Robotic Assisted Procedure of Trunk Region, Percutaneous Endoscopic Approach	[38]
	**8E0W7CZ**	Robotic Assisted Procedure of Trunk Region, Via Natural or Artificial Opening	[38]
	**8E0W8CZ**	Robotic Assisted Procedure of Trunk Region, Via Natural or Artificial Opening Endoscopic	[38]

© 2022 Ramkumar N et al. *JAMA Network Open*.

Table C5. ICD 10 diagnosis and procedure codes for complications following cancer-directed surgery for localized colon cancer. Derived from a modified partial Iezzoni et al algorithm.[25,26]

Code	Description
Post-operative complications relating to urinary tract anatomy (renal failure) (M)	
Diagnostic	
**N17.0**	Acute kidney failure with tubular necrosis
**N17.1**	Acute kidney failure with acute cortical necrosis
**N17.2**	Acute kidney failure with medullary necrosis
**N17.8**	Other acute kidney failure
**N17.9**	Acute kidney failure, unspecified
Procedural	
**5A1.D00Z**	Performance of urinary filtration, single
**5A1.D60Z**	Performance of urinary filtration, multiple
Post-operative pulmonary compromise (M)	
Diagnostic	
**J96.00**	Acute respiratory failure, unsp w hypoxia or hypercapnia
**J96.01**	Acute respiratory failure with hypoxia
**J96.02**	Acute respiratory failure with hypercapnia
**J96.20**	Acute and chr resp failure, unsp w hypoxia or hypercapnia
**J96.21**	Acute and chronic respiratory failure with hypoxia
**J96.22**	Acute and chronic respiratory failure with hypercapnia
**J96.90**	Respiratory failure, unsp, unsp w hypoxia or hypercapnia
**J96.91**	Respiratory failure, unspecified with hypoxia
**J96.92**	Acute and chr resp failure, unsp w hypoxia or hypercapnia
**J80**	Acute respiratory distress syndrome
Procedural	
**0BH.17EZ**	Insertion of Endotracheal Airway into Trachea, Via Natural or Artificial Opening
**0BH.18EZ**	Insertion of Endotracheal Airway into Trachea, Via Natural or Artificial Opening Endoscopic
**0B7.17DZ**	Dilation of Trachea with Intraluminal Device, Via Natural or Artificial Opening
**0B7.18DZ**	Dilation of Trachea with Intraluminal Device, Via Natural or Artificial Opening Endoscopic
**0BH.072Z**	Insertion of Monitoring Device into Tracheobronchial Tree, Via Natural or Artificial Opening
**0BH.073Z**	Insertion of Infusion Device into Tracheobronchial Tree, Via Natural or Artificial Opening
**0BH.07DZ**	Insertion of Intraluminal Device into Tracheobronchial Tree, Via Natural or Artificial Opening
**0BH.172Z**	Insertion of Monitoring Device into Trachea, Via Natural or Artificial Opening
**0BH.182Z**	Insertion of Monitoring Device into Trachea, Via Natural or Artificial Opening Endoscopic
**0BH.K72Z**	Insertion of Monitoring Device into Right Lung, Via Natural or Artificial Opening
**0BH.K73Z**	Insertion of Infusion Device into Right Lung, Via Natural or Artificial Opening

© 2022 Ramkumar N et al. *JAMA Network Open*.

**0BH.K82Z**	Insertion of Monitoring Device into Right Lung, Via Natural or Artificial Opening Endoscopic
**0BH.K83Z**	Insertion of Infusion Device into Right Lung, Via Natural or Artificial Opening Endoscopic
**0BH.L72Z**	Insertion of Monitoring Device into Left Lung, Via Natural or Artificial Opening
**0BH.L73Z**	Insertion of Infusion Device into Left Lung, Via Natural or Artificial Opening
**0BH.L82Z**	Insertion of Monitoring Device into Left Lung, Via Natural or Artificial Opening Endoscopic
**0BH.L83Z**	Insertion of Infusion Device into Left Lung, Via Natural or Artificial Opening Endoscopic
**0WH.Q73Z**	Insertion of Infusion Device into Respiratory Tract, Via Natural or Artificial Opening
**0WH.Q7YZ**	Insertion of Other Device into Respiratory Tract, Via Natural or Artificial Opening
**5A1.935Z**	Respiratory Ventilation, Less than 24 Consecutive Hours
**5A1.945Z**	Respiratory Ventilation, 24-96 Consecutive Hours
**5A1.955Z**	Respiratory Ventilation, Greater than 96 Consecutive Hours
Post-operative acute myocardial infarction (M)	
Diagnostic	
**I21.09**	ST elevation (STEMI) myocardial infarction involving other coronary artery of anterior wall
**I22.0**	Subsequent ST elevation (STEMI) myocardial infarction of anterior wall
**I21.09**	ST elevation (STEMI) myocardial infarction involving other coronary artery of anterior wall
**I21.01**	ST elevation (STEMI) myocardial infarction involving left main coronary artery
**I21.02**	ST elevation (STEMI) myocardial infarction involving left anterior descending coronary artery
**I22.1**	Subsequent ST elevation (STEMI) myocardial infarction of inferior wall
**I21.11**	ST elevation (STEMI) myocardial infarction involving right coronary artery
**I21.19**	ST elevation (STEMI) myocardial infarction involving other coronary artery of inferior wall
**I21.29**	ST elevation (STEMI) myocardial infarction involving other sites
**I22.8**	Subsequent ST elevation (STEMI) myocardial infarction of other sites
**I21.4**	Non-ST elevation (NSTEMI) myocardial infarction
**I22.2**	Subsequent non-ST elevation (NSTEMI) myocardial infarction
**I21.21**	ST elevation (STEMI) myocardial infarction involving left circumflex coronary artery
**I21.3**	ST elevation (STEMI) myocardial infarction of unspecified site
**I22.9**	Subsequent ST elevation (STEMI) myocardial infarction of unspecified site
Post-operative pneumonia (M)	
Diagnostic	
**J13**	Pneumonia due to Streptococcus pneumoniae
**J18.1**	Lobar pneumonia, unspecified organism
**J15.0**	Pneumonia due to Klebsiella pneumoniae
**J15.1**	Pneumonia due to Pseudomonas
**J14**	Pneumonia due to Hemophilus influenzae
**J15.4**	Pneumonia due to other streptococci
**J15.3**	Pneumonia due to streptococcus, group B
**J15.20**	Pneumonia due to staphylococcus, unspecified
**J15.211**	Pneumonia due to Methicillin susceptible Staphylococcus aureus

© 2022 Ramkumar N et al. *JAMA Network Open*.

**J15.212**	Pneumonia due to Methicillin resistant Staphylococcus aureus
**J15.29**	Pneumonia due to other staphylococcus
**J15.8**	Pneumonia due to other specified bacteria
**J15.5**	Pneumonia due to Escherichia coli
**J15.6**	Pneumonia due to other aerobic Gram-negative bacteria
**A48.1**	Legionnaires' disease
**J15.9**	Unspecified bacterial pneumonia
**J15.7**	Pneumonia due to Mycoplasma pneumoniae
**J16.0**	Chlamydial pneumonia
**J16.8**	Pneumonia due to other specified infectious organisms
**B25.0**	Cytomegaloviral pneumonitis
**A22.1**	Pulmonary anthrax
**B44.0**	Invasive pulmonary aspergillosis
**J17**	Pneumonia in diseases classified elsewhere
**B77.81**	Ascariasis pneumonia
**J18.0**	Bronchopneumonia, unspecified organism
**J69.0**	Pneumonitis due to inhalation of food and vomit
Venous thrombosis and pulmonary embolism (M)	
Diagnostic	
**I26.99**	Other pulmonary embolism without acute cor pulmonale
**I26.02**	Saddle embolus of pulmonary artery with acute cor pulmonale
**I26.92**	Saddle embolus of pulmonary artery without acute cor pulmonale
**I26.09**	Other pulmonary embolism with acute cor pulmonale
**I80.10**	Phlebitis and thrombophlebitis of unspecified femoral vein
**I80.11**	Phlebitis and thrombophlebitis of right femoral vein
**I80.12**	Phlebitis and thrombophlebitis of left femoral vein
**I80.13**	Phlebitis and thrombophlebitis of femoral vein, bilateral
**I80.201**	Phlebitis and thrombophlebitis of unspecified deep vessels of right lower extremity
**I80.202**	Phlebitis and thrombophlebitis of unspecified deep vessels of left lower extremity
**I80.203**	Phlebitis and thrombophlebitis of unspecified deep vessels of lower extremities, bilateral
**I80.209**	Phlebitis and thrombophlebitis of unspecified deep vessels of unspecified lower extremity
**I80.221**	Phlebitis and thrombophlebitis of right popliteal vein
**I80.222**	Phlebitis and thrombophlebitis of left popliteal vein
**I80.223**	Phlebitis and thrombophlebitis of popliteal vein, bilateral
**I80.229**	Phlebitis and thrombophlebitis of unspecified popliteal vein
**I80.231**	Phlebitis and thrombophlebitis of right tibial vein
**I80.232**	Phlebitis and thrombophlebitis of left tibial vein
**I80.233**	Phlebitis and thrombophlebitis of tibial vein, bilateral
**I80.239**	Phlebitis and thrombophlebitis of unspecified tibial vein
**I80.291**	Phlebitis and thrombophlebitis of other deep vessels of right lower extremity
**I80.292**	Phlebitis and thrombophlebitis of other deep vessels of left lower extremity
**I80.293**	Phlebitis and thrombophlebitis of other deep vessels of lower extremity, bilateral
**I80.299**	Phlebitis and thrombophlebitis of other deep vessels of unspecified lower extremity
**I80.3**	Phlebitis and thrombophlebitis of lower extremities, unspecified
**I80.211**	Phlebitis and thrombophlebitis of right iliac vein
**I80.212**	Phlebitis and thrombophlebitis of left iliac vein

© 2022 Ramkumar N et al. *JAMA Network Open*.

**I80.213**	Phlebitis and thrombophlebitis of iliac vein, bilateral
**I80.219**	Phlebitis and thrombophlebitis of unspecified iliac vein
**I82.621**	Acute embolism and thrombosis of deep veins of right upper extremity
**I82.622**	Acute embolism and thrombosis of deep veins of left upper extremity
**I82.623**	Acute embolism and thrombosis of deep veins of upper extremity, bilateral
**I82.629**	Acute embolism and thrombosis of deep veins of unspecified upper extremity
**I82.601**	Acute embolism and thrombosis of unspecified veins of right upper extremity
**I82.602**	Acute embolism and thrombosis of unspecified veins of left upper extremity
**I82.603**	Acute embolism and thrombosis of unspecified veins of upper extremity, bilateral
**I82.609**	Acute embolism and thrombosis of unspecified veins of unspecified upper extremity
**I82.A11**	Acute embolism and thrombosis of right axillary vein
**I82.A12**	Acute embolism and thrombosis of left axillary vein
**I82.A13**	Acute embolism and thrombosis of axillary vein, bilateral
**I82.A19**	Acute embolism and thrombosis of unspecified axillary vein
**I82.B11**	Acute embolism and thrombosis of right subclavian vein
**I82.B12**	Acute embolism and thrombosis of left subclavian vein
**I82.B13**	Acute embolism and thrombosis of subclavian vein, bilateral
**I82.B19**	Acute embolism and thrombosis of unspecified subclavian vein
**I82.C11**	Acute embolism and thrombosis of right internal jugular vein
**I82.C12**	Acute embolism and thrombosis of left internal jugular vein
**I82.C13**	Acute embolism and thrombosis of internal jugular vein, bilateral
**I82.C19**	Acute embolism and thrombosis of unspecified internal jugular vein
**I82.210**	Acute embolism and thrombosis of superior vena cava
**I82.290**	Acute embolism and thrombosis of other thoracic veins
**I82.890**	Acute embolism and thrombosis of other specified veins
**I82.90**	Acute embolism and thrombosis of unspecified vein
Procedural	
**02H.V3DZ**	Insertion of Intraluminal Device into Superior Vena Cava, Percutaneous Approach
**02H.V4DZ**	Insertion of Intraluminal Device into Superior Vena Cava, Percutaneous Endoscopic Approach
**06H.03DZ**	Insertion of Intraluminal Device into Inferior Vena Cava, Percutaneous Approach
**06H.04DZ**	Insertion of Intraluminal Device into Inferior Vena Cava, Percutaneous Endoscopic Approach
Post-procedural hemorrhage or hematoma (S)	
Diagnostic	
**T88.8XXA**	Other specified complications of surgical and medical care, not elsewhere classified, initial encounter
**I97.621**	Postprocedural hematoma of a circulatory system organ or structure following other procedure
**K91.870**	Postprocedural hematoma of a digestive system organ or structure following a digestive system procedure
**K91.871**	Postprocedural hematoma of a digestive system organ or structure following other procedure
**N99.840**	Postprocedural hematoma of a genitourinary system organ or structure following a genitourinary system procedure
**N99.841**	Postprocedural hematoma of a genitourinary system organ or structure following other procedure

© 2022 Ramkumar N et al. *JAMA Network Open*.

**G97.62**	Postprocedural hematoma of a nervous system organ or structure following other procedure
**J95.861**	Postprocedural hematoma of a respiratory system organ or structure following other procedure
**E89.821**	Postprocedural hematoma of an endocrine system organ or structure following other procedure
**H95.52**	Postprocedural hematoma of ear and mastoid process following other procedure
**H59.343**	Postprocedural hematoma of eye and adnexa following other procedure, bilateral
**H59.342**	Postprocedural hematoma of left eye and adnexa following other procedure
**H59.341**	Postprocedural hematoma of right eye and adnexa following other procedure
**L76.32**	Postprocedural hematoma of skin and subcutaneous tissue following other procedure
**D78.32**	Postprocedural hematoma of the spleen following other procedure
**H59.349**	Postprocedural hematoma of unspecified eye and adnexa following other procedure
**I97.620**	Postprocedural hemorrhage of a circulatory system organ or structure following other procedure
**K91.840**	Postprocedural hemorrhage of a digestive system organ or structure following a digestive system procedure
**K91.841**	Postprocedural hemorrhage of a digestive system organ or structure following other procedure
**N99.821**	Postprocedural hemorrhage of a genitourinary system organ or structure following other procedure
**M96.831**	Postprocedural hemorrhage of a musculoskeletal structure following other procedure
**G97.52**	Postprocedural hemorrhage of a nervous system organ or structure following other procedure
**J95.831**	Postprocedural hemorrhage of a respiratory system organ or structure following other procedure
**E89.811**	Postprocedural hemorrhage of an endocrine system organ or structure following other procedure
**H59.323**	Postprocedural hemorrhage of eye and adnexa following other procedure, bilateral
**H59.322**	Postprocedural hemorrhage of left eye and adnexa following other procedure
**H59.321**	Postprocedural hemorrhage of right eye and adnexa following other procedure
**H59.329**	Postprocedural hemorrhage of unspecified eye and adnexa following other procedure
**H95.42**	Postprocedural hemorrhage of ear and mastoid process following other procedure
**L76.22**	Postprocedural hemorrhage of skin and subcutaneous tissue following other procedure
**D78.22**	Postprocedural hemorrhage of the spleen following other procedure
Wound infection (S)	
Diagnostic	
**T79.8XXA**	Other early complications of trauma, initial encounter
**T81.30XA**	Disruption of wound, unspecified, initial encounter
**T81.32XA**	Disruption of internal operation (surgical) wound, not elsewhere classified, initial encounter
**T81.31XA**	Disruption of external operation (surgical) wound, not elsewhere classified, initial encounter
**T81.33XA**	Disruption of traumatic injury wound repair, initial encounter
**T81.4XXA**	Infection following a procedure, initial encounter
Procedural	
**0W9.F30Z**	Drainage of Abdominal Wall with Drainage Device, Percutaneous Approach

© 2022 Ramkumar N et al. *JAMA Network Open*.

**0W9.F3ZZ**	Drainage of Abdominal Wall, Percutaneous Approach
**0W9.F40Z**	Drainage of Abdominal Wall with Drainage Device, Percutaneous Endoscopic Approach
**0W9.F4ZZ**	Drainage of Abdominal Wall, Percutaneous Endoscopic Approach
**0W9.G30Z**	Drainage of Peritoneal Cavity with Drainage Device, Percutaneous Approach
**0W9.G3ZX**	Drainage of Peritoneal Cavity, Percutaneous Approach, Diagnostic
**0W9.G3ZZ**	Drainage of Peritoneal Cavity, Percutaneous Approach
**0W9.G40Z**	Drainage of Peritoneal Cavity with Drainage Device, Percutaneous Endoscopic Approach
**0W9.G4ZZ**	Drainage of Peritoneal Cavity, Percutaneous Endoscopic Approach
**0W9.H30Z**	Drainage of Retroperitoneum with Drainage Device, Percutaneous Approach
**0W9.H3ZZ**	Drainage of Retroperitoneum, Percutaneous Approach
**0W9.J30Z**	Drainage of Pelvic Cavity with Drainage Device, Percutaneous Approach
**0W9.J3ZZ**	Drainage of Pelvic Cavity, Percutaneous Approach
Dementia/Delirium (M)	
Diagnostic	
**F05**	Delirium due to known physiological condition
**F06.8**	Other specified mental disorders due to known physiological condition
Anastomotic Leak (S)	
Diagnostic	
**Y83.2**	Surgical operation with anastomosis, bypass or graft as the cause of abnormal reaction of the patient, or of later complication, without mention of misadventure at the time of the procedure
**Y83.3**	Surgical operation with formation of external stoma as the cause of abnormal reaction of the patient, or of later complication, without mention of misadventure at the time of the procedure
Abdominal Abscess (S)	
Diagnostic	
**K65.1**	Peritoneal abscess
**K68.19**	Other retroperitoneal abscess
**K68.11**	Postprocedural retroperitoneal abscess
**K68.12**	Psoas muscle abscess
**K63.0**	Abscess of intestine

© 2022 Ramkumar N et al. *JAMA Network Open*.

eFigure. Time to Receipt of Surgery in the Overall Cohort (N = 57 710) and by Rurality

(A) Kaplan-Meier time-to-event graph and (B) forest plot of adjusted hazard ratios for receipt of surgery estimated under the Cox proportional hazards model.

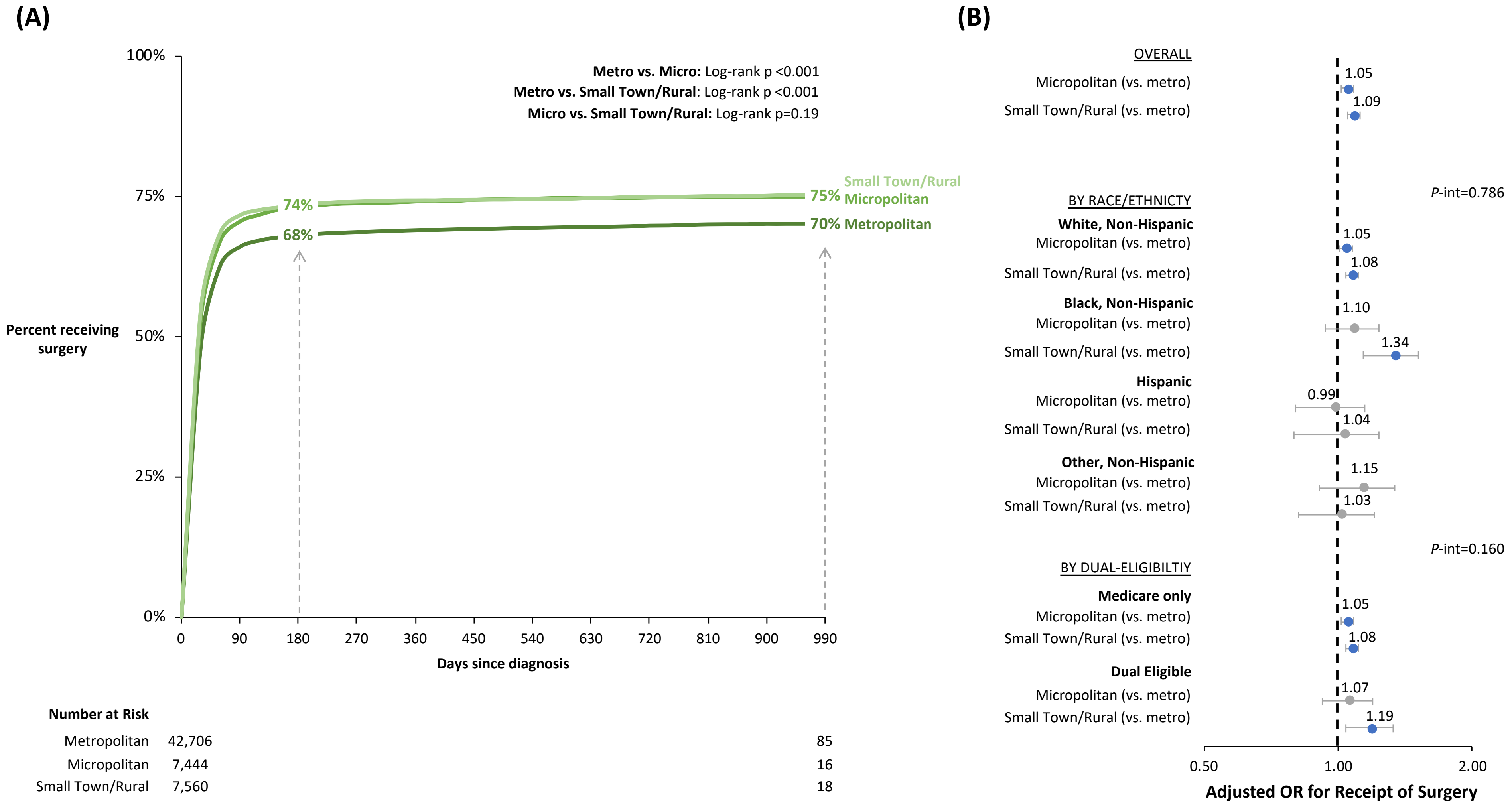

* Models were adjusted for age, sex, race, dual eligibility, area deprivation index, cancer side, diabetes, congestive heart failure, chronic obstructive pulmonary disease, previous myocardial infarction, stroke or transient ischemic attack, end-stage renal disease, and CMS-HCC score. Adjusted odds ratios for all covariates in the model are available in Table 2.

© 2022 Ramkumar N et al. *JAMA Network Open*.

eTable. Patient Characteristics and Surgical Management Measures Among the Surgical Cohort by Rurality and by Race and Ethnicity

Patient Region	Measure	White, Non-Hispanic	Black, Non-Hispanic	Hispanic	Other, Non-Hispanic	p-value
Metropolitan	**N in group**	**23,164**	**1,875**	**1,125**	**1,141**	
	**Median ADI rank (IQR)**	38.0 (20.0-59.0)	62.0 (35.0-84.0)	49.0 (24.0-76.0)	22.0 (10.0-49.0)	<0.001
	**Full Dual Eligible,** N (%)	738 (3.2%)	240 (12.8%)	265 (23.6%)	276 (24.2%)	<0.001
	**Median Days to Surgery (IQR)**	15.0 (2.0-30.0)	14.0 (2.0-34.0)	14.0 (3.0-33.0)	17.0 (3.0-33.0)	0.034
	**Rurality of Surgical Hospital,** N (%)					<0.001
	Metropolitan	22,201 (97.6%)	1,783 (98.0%)	1,099 (99.3%)	1,117 (98.8%)	
	Micropolitan	401 (1.8%)	--	<11	<11	
	Small Town/Rural	147 (0.6%)	<11	<11	<11	
	**Median miles travelled (IQR)**	7.3 (3.5-14.7)	5.9 (3.0-11.0)	6.2 (3.1-12.1)	6.4 (3.2-11.8)	<0.001
	**Non-elective Surgery,** N (%)	2,539 (11.0%)	196 (10.5%)	132 (11.7%)	126 (11.0%)	0.76
	**90-day Surgical Complications,** N (%)	2,635 (11.4%)	223 (11.9%)	134 (11.9%)	123 (10.8%)	0.76
Micropolitan	**N in group**	**4,607**	**237**	**131**	**125**	
	**Median ADI rank (IQR)**	62.0 (46.0-77.0)	84.0 (69.0-92.0)	65.6 (48.0-80.0)	51.0 (37.0-69.0)	<0.001
	**Full Dual Eligible,** N (%)	199 (4.3%)	51 (21.5%)	23 (17.6%)	12 (9.6%)	<0.001
	**Median Days to Surgery (IQR)**	14.0 (2.0-29.0)	9.0 (1.0-29.0)	13.0 (2.0-35.0)	17.0 (2.0-33.0)	0.32
	**Rurality of Surgical Hospital,** N (%)					0.037
	Metropolitan	1,874 (41.6%)	85 (36.6%)	67 (52.8%)	59 (48.0%)	
	Micropolitan	2,523 (56.0%)	--	--	--	
	Small Town/Rural	109 (2.4%)	<11	<11	<11	
	**Median miles travelled (IQR)**	14.8 (0.0-36.3)	15.4 (0.0-34.1)	18.6 (0.0-46.5)	18.3 (1.3-54.2)	0.23
	**Non-elective Surgery,** N (%)	624 (13.5%)	31 (13.1%)	17 (13.0%)	19 (15.2%)	0.95
	**90-day Surgical Complications,** N (%)	559 (12.1%)	32 (13.5%)	33 (25.2%)	17 (13.6%)	<0.001
Small Town/Rural	**N in group**	**4,834**	**222**	**88**	**142**	
	**Median ADI rank (IQR)**	67.0 (52.1-79.2)	83.0 (70.0-92.0)	75.5 (60.8-89.0)	73.8 (55.0-86.0)	<0.001
	**Full Dual Eligible,** N (%)	257 (5.3%)	44 (19.8%)	16 (18.2%)	26 (18.3%)	<0.001
	**Median Days to Surgery (IQR)**	13.0 (2.0-28.0)	8.0 (1.0-25.0)	15.5 (2.0-35.5)	14.0 (3.0-33.0)	0.038
	**Rurality of Surgical Hospital,** N (%)					0.003
	Metropolitan	2,714 (57.2%)	120 (56.1%)	63 (72.4%)	86 (62.8%)	
	Micropolitan	1,023 (21.6%)	61 (28.5%)	13 (14.9%)	32 (23.4%)	
	Small Town/Rural	1,004 (21.2%)	33 (15.4%)	11 (12.6%)	19 (13.9%)	
	**Median miles travelled (IQR)**	31.7 (17.0-54.8)	31.3 (18.0-49.9)	43.0 (26.7-74.3)	52.8 (28.3-97.0)	<0.001
	**Non-elective Surgery,** N (%)	699 (14.5%)	40 (18.0%)	14 (15.9%)	19 (13.4%)	0.49
	**90-day Surgical Complications,** N (%)	592 (12.2%)	35 (15.8%)	14 (15.9%)	27 (19.0%)	0.033

© 2022 Ramkumar N et al. *JAMA Network Open*.

ADI= Area Deprivation Index, IQR=interquartile range. There are small proportions of missing data (1.9%) in rurality of surgical hospital and median miles travelled.

© 2022 Ramkumar N et al. *JAMA Network Open*.
